# *Paraphocaeicola brunensis* gen. nov., sp. nov., Carrying Two Variants of *nimB* Resistance Gene from Bacteroides fragilis, and *Caecibacteroides pullorum* gen. nov., sp. nov., Two Novel Genera Isolated from Chicken Caeca

**DOI:** 10.1128/spectrum.01954-21

**Published:** 2022-02-16

**Authors:** Stanislava Kralova, Lenka Davidova-Gerzova, Adam Valcek, Matej Bezdicek, Ivan Rychlik, Veronika Rezacova, Alois Cizek

**Affiliations:** a CEITEC VFU, University of Veterinary Sciences Brno, Brno, Czech Republic; b Department of Experimental Biology, Czech Collection of Microorganisms, Faculty of Science, Masaryk University, Brno, Czech Republic; c Microbial Resistance and Drug Discovery, VIB-VUB Center for Structural Biology, VIB, Flanders Institute for Biotechnology, Brussels, Belgium; d Structural Biology Brussels, Vrije Universiteit Brussel (VUB), Brussels, Belgium; e Department of Internal Medicine – Hematology and Oncology, University Hospital, Brno, Czech Republic; f Department of Internal Medicine – Hematology and Oncology, Masaryk University, Brno, Czech Republic; g Department of Bacteriology, Veterinary Research Institute, v. V. I., Brno, Czech Republic; h The Institute of Chemistry and Technology of Environmental Protection, Faculty of Chemistry, Brno University of Technology, Brno, Czech Republic; i Institute of Infectious Diseases and Microbiology, Faculty of Veterinary Medicine Brno, University of Veterinary Sciences Brno, Brno, Czech Republic; University of Minnesota

**Keywords:** phylogenomics, polyphasic taxonomy, *Paraphocaeicola brunensis* gen. nov., sp. nov., *Caecibacteroides pullorum* gen. nov., sp. nov., *Bacteroidaceae*, metronidazole resistance, *nimB* gene

## Abstract

Three difficult-to-cultivate, strictly anaerobic strains, AN20^T^, AN421^T^, and AN502, were analyzed within a project studying possible probiotics for newly hatched chickens. Phylogenetic analyses showed that strains AN20^T^, AN421^T^, and AN502 formed two well-separated phylogenetic lineages in all phylogenetic and phylogenomic trees comprising members of the family *Bacteroidaceae*. Comparison to reference genomes of type species Bacteroides fragilis NCTC 9343^T^, Phocaeicola abscessus CCUG 55929^T^, and Capsularis zoogleoformans ATCC 33285^T^ showed low relatedness based on the calculated genome-to-genome distance and orthologous average nucleotide identity. Analysis of fatty acid profiles showed iso-C_15:0_, anteiso-C_15:0_, C_16:0_, C_18:1_ *ω9c*, and iso-C_17:0_ 3OH as the major fatty acids for all three strains and additionally C_16:0_ 3OH for AN421^T^ and AN502. A specific combination of respiratory quinones different from related taxa was found in analyzed strains, MK-5 plus MK-11 in strain AN20^T^ and MK-5 plus MK-10 in strains AN421^T^ and AN502. Strains AN421^T^ and AN502 harbor complete CRISPR loci with CRISPR array, type II-C, accompanied by a set of *cas* genes (*cas9*, *cas1*, and *cas2*) in close proximity. Interestingly, strain AN20^T^ was found to harbor two copies of *nimB* gene with >95% similarity to *nimB* of B. fragilis, suggesting a horizontal gene transfer between these taxa. In summary, three isolates characterized in this study represent two novel species, which we proposed to be classified in two novel genera of the family *Bacteroidaceae*, for which the names *Paraphocaeicola brunensis* sp. nov. (AN20^T^ = CCM 9041^T^ = DSM 111154^T^) and *Caecibacteroides pullorum* sp. nov. (AN421^T^= CCM 9040^T^ = DSM 111155^T^) are proposed.

**IMPORTANCE** This study represents follow-up research on three difficult-to-cultivate anaerobic isolates originally isolated within a project focused on strains that are able to stably colonize newly hatched chickens, thus representing possible probiotics. This project is exceptional in that it successfully isolates several miscellaneous strains that required modified and richly supplemented anaerobic media, as information on many gut-colonizing bacteria is based predominantly on metagenomic studies. Superior colonization of newly hatched chickens by *Bacteroides* spp., *Phocaeicola* spp., or related taxa can be considered of importance for development of future probiotics. Although different experiments can also be performed with provisionally characterized isolates, precise taxonomical definition is necessary for subsequent broad communication. The aim of this study is therefore to thoroughly characterize these isolates that represent novel genera and precisely determine their taxonomic position among related taxa to facilitate further research and communication involving these strains.

## INTRODUCTION

The family *Bacteroidaceae* in the order *Bacteroidales* underwent numerous taxonomic changes in recent years that were driven mostly by introducing genomic insights into bacterial classification. Advances of next-generation sequencing and comparative genomics based on whole-genome data significantly improved classification within numerous taxa, including exclusion of multiple genera from the family *Bacteroidaceae* and establishment of novel monophyletic families *Prevotellaceae*, *Porphyromonadaceae*, and *Rikenellaceae* (1). The genus *Bacteroides* was only recently subjected to a large genomic study which resulted in further taxonomic reclassifications within this genus and separation of its members into two genera, *Bacteroides* and *Phocaeicola*, with 10 previously described *Bacteroides* spp. assigned to the later genus (2). The family *Bacteroidaceae* currently comprises only four genera, out of which two, *Bacteroides* and *Phocaeicola*, are of clinical importance in human or veterinary medicine, which is defined by their presence in gut microbiota (3). Members of both genera are important gut commensals capable of degradation and fermentation of mucin or complex polysaccharides of plant origin (4). However, outside the intestinal tract, *Bacteroides* spp. and *Phocaeicola* spp. can be involved in various pathogenic processes (5). Clinical importance grows with horizontal gene transfer ability of *Bacteroides* spp. and their relatives, especially through conjugation, which contributes to increase in antibiotic resistance of these bacteria (6). Prevalence of tetracycline resistance (*tetQ*) almost tripled among *Bacteroides* spp. in the past 30 years, and resistance to erythromycin (*ermF*, *ermG*) rose from below 2% up to 23% (7). The resistance to 5-nitroimidazole (*nim* genes)-derived antibiotics is particularly of concern because these drugs are routinely prescribed for treatment of anaerobic infections (8).

The importance and prevalence of anaerobic bacteria among gut microbiota are often based on metagenomic studies, due to their complicated isolation. However, due to the presence of *Bacteroides* spp. and *Phocaeicola* spp. in gut microbiota and superior efficiency in colonization of newly hatched chickens, these and related bacterial species can be considered future probiotics (9). Although different experiments can also be performed with provisionally characterized isolates, precise taxonomical definition is necessary for subsequent broad communication. Therefore, these three strains isolated from gastrointestinal tracts of chickens (10) are thoroughly characterized in the present study in order to precisely determine their taxonomic position.

## RESULTS AND DISCUSSION

### Isolation of anaerobic strains.

The strain AN20^T^ characterized in this study was isolated within a previous study focused on cultures of chicken gut microbiota and predictions of their functions (10). Strains AN421^T^ and AN502 were isolated from the chicken ceca in follow-up experiments (11).

### Phylogenetics.

At first, the 16S rRNA gene sequences were extracted from whole genomes as well as sequenced separately for identity confirmation. The nearly complete 16S rRNA gene sequences of AN20^T^ (1,527 bp, accession no. MT894137), AN421^T^ (1,525 bp, accession no. MT894142), and AN502 (1,525 bp, accession no. MT894135) were identified using the EzBioCloud database (12). Based on the initial identification of the 16S rRNA sequences, members of the genera *Bacteroides*, *Capsularis*, and *Phocaeicola* were assigned as the closest related species, however with significantly low 16S rRNA gene sequence similarities ≤93.5%. The closest related phylogenetic neighbors for AN20^T^ were Bacteroides uniformis ATCC 8492^T^ (90.34%) and Capsularis zoogleoformans ATCC 33285^T^ (90.13%). The closest relatives identified for AN421^T^ and AN502 were Bacteroides eggerthii (93.43 and 93.50%, respectively), Bacteroides gallinarum JCM 13658^T^ (93.23 and 93.16%, respectively), and Bacteroides uniformis ATCC 8492^T^ (93.23 and 93.09%, respectively). The pairwise comparison of 16S rRNA gene sequences between AN421^T^ and AN502 strains revealed 99.61% sequence identity and 90.74% 16S rRNA gene sequence similarity to the strain AN20^T^. Sequence similarities values between the three strains and the closest related species were all well below the threshold value 98.65% suggested for species delineation (13) and also below the 95% recommended for genera delineation (14).

**FIG 1:**
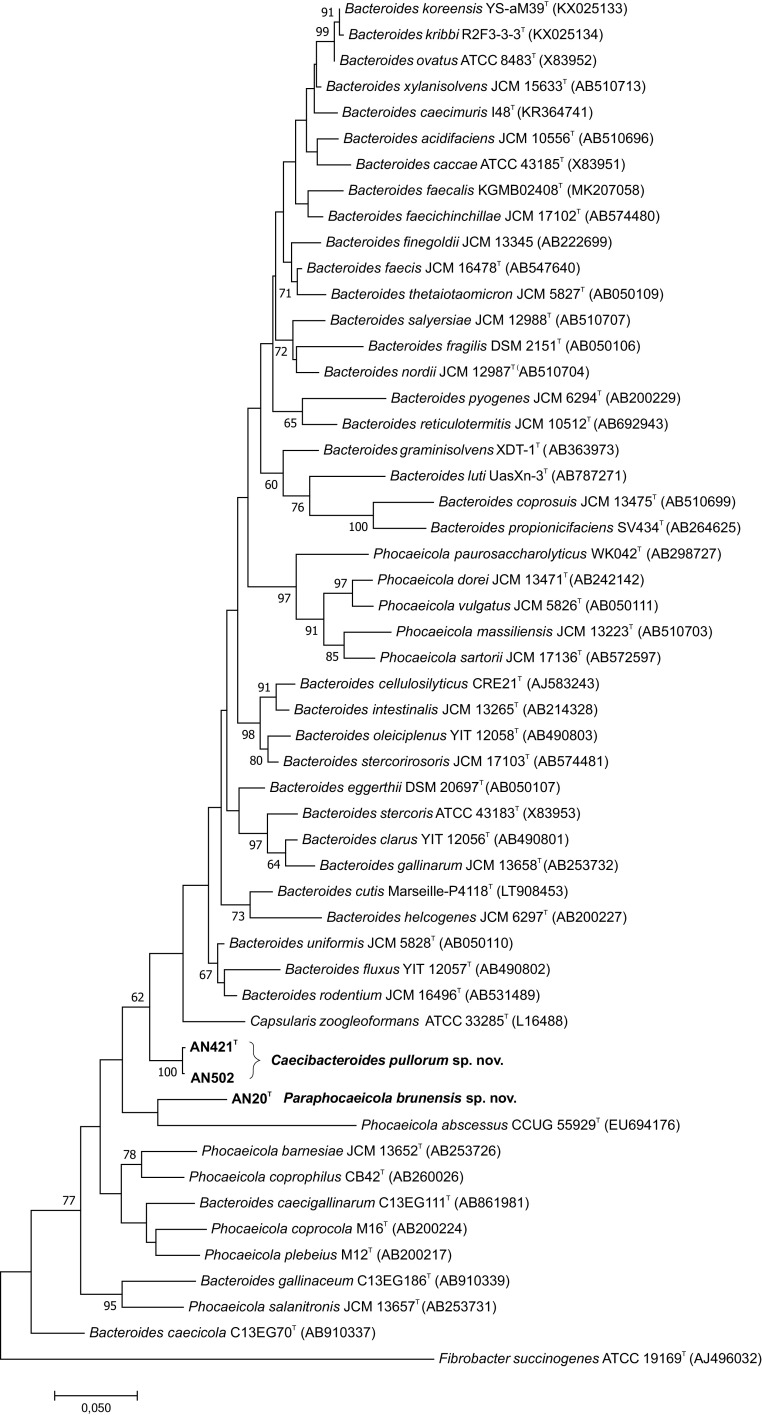
Phylogenetic tree based on comparison of 16S rRNA gene sequences showing the phylogenetic position of *Pseudophocaeicola brunensis* sp. nov. (AN20^T^) and *Caecibacteroides pullorum* sp. nov. (AN421^T^, AN502) among the closest relatives within the family *Bacteroidaceae*. The evolutionary history was inferred by using the maximum-likelihood method based on the Kimura two-parameter model. All positions with less than 95% site coverage were eliminated. Bootstrap probability values (percentages of 1,000 tree replications) greater than 60% are indicated at branch points. Fibrobacter succinogenes ATCC 19169^T^ (AJ496032) was used as an outgroup. Bar, 0.05 substitutions per nucleotide position.

To comply with the current valid taxonomy, this paper employs in further analyses genus *Capsularis* as a separate genus containing one species, *C. zoogleoformans*, reflecting that this genus still retains its validity and standing in the nomenclature (LPNS, https://www.bacterio.net/) and has not yet been officially reclassified. This species is, however, recognized in most literature relating to anaerobes as Prevotella zoogleoformans and/or Bacteroides zoogleoformans. Although reclassification of this species is not yet officially resolved, it is clear from recent research applying whole genomic data that *C. zoogleoformans* should be reclassified to the genus *Bacteroides* as *B. zoogleoformans* (2). The phylogenetic trees from all validly described species of the genera *Bacteroides*, *Capsularis*, and recently-emended genus *Phocaeicola* were reconstructed to allocate phylogenetic position of the three analyzed strains in regard to these taxa. In the maximum-likelihood (ML) tree, strain AN20^T^ formed a line distinctly related to Phocaeicola abscessus CCUG 55929^T^, while strains AN421^T^ and AN502 formed a monophyletic lineage placed between the genera *Bacteroides*, *Capsularis*, and *Phocaeicola* (Fig. 1). Bayesian phylogeny showed two monophyletic clades formed within the family *Bacteroidaceae*. The first one comprised all three analyzed strains characterized in this study, forming a monophyletic cluster along with all validly named *Phocaeicola* spp. and three *Bacteroides* spp. that were also recently suggested to be reclassified to the genus *Phocaeicola* (2). The second clade comprised members of the genus *Bacteroides* and *C. zoogleoformans* ATCC 33285^T^ forming a distinct lineage within this clade (Fig. S1 in the supplemental material). Phylogenetic analysis based on the 16S rRNA genes clearly showed that strains AN20^T^, AN421^T^, and AN502 belong to the family *Bacteroidaceae* and further suggested that their phylogenetic position is distant from that of other related species from this family.

### Phylogenomics.

**FIG 2:**
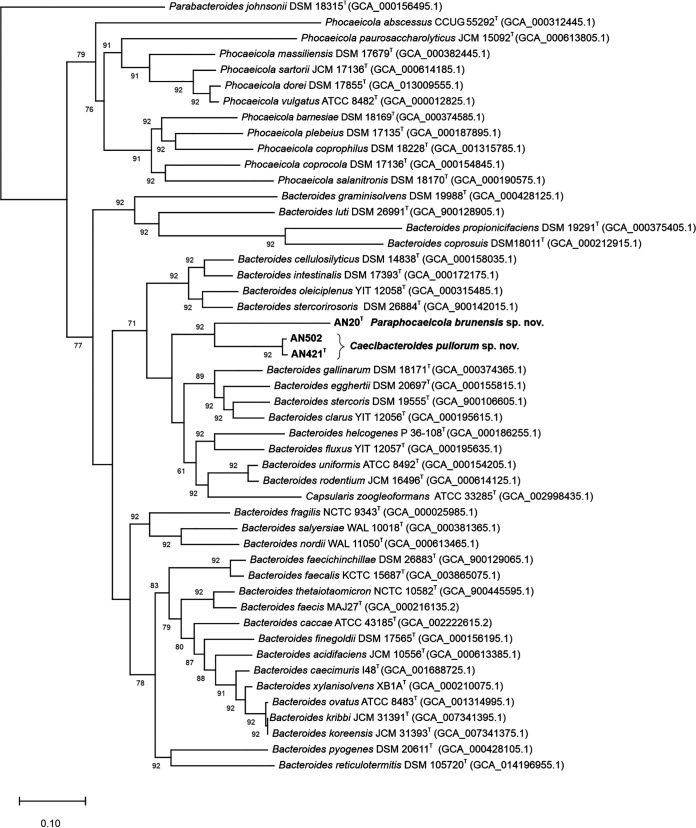
Phylogenetic tree reconstructed by an up-to-date bacterial core gene (UBCG) set consisting of 92 genes. Gene support index (GGI) values greater than 60% are indicated on the branches. Parabacteroides johnsonii DSM 18315^T^ (GCA_000156495.1) was used as an outgroup. Bar, 0.5 substitutions per nucleotide position.

To obtain a phylogenetic assignment more accurate than that of the 16S rRNA-based phylogenesis, the phylogenetic trees based on 92 concatenated core genes were calculated using UBCG pipeline with default parameters (15). The whole/draft genome sequences of the reference strains, including only validly described type strains of *Bacteroides* spp., *Phocaeicola* spp., and *Capsularis* sp., were retrieved from the NCBI database. Strains AN20^T^, AN421^T^, and AN502 were placed in a monophyletic clade within the cluster comprising *Bacteroides* spp. and *C. zoogleoformans* (Fig. 2). The UBCG tree suggested that AN20^T^ is more closely related to AN421^T^ and AN502 and to genera *Bacteroides* and *Capsularis* than to the genus *Phocaeicola*, contrary to the Bayesian phylogeny.

To further refine taxonomic assignment of the three strains based on whole-genome data, the draft genome sequences were submitted to the Type Strain Genome Server (TYGS) and Microbial Genome atlas MiGA tools. Strain AN20^T^ created a separated line in the MiGA evolutionary tree, in the closest phylogenetic distance to Bacteroides uniformis DSM 6597^T^ and Bacteroides rodentium JCM 16494^T^. Strain AN421^T^ and strain AN502 formed similarly separated lines in the MiGA evolutionary tree with “*Bacteroides togonis*” Marseille-P1366 (GCA_900130135), “*Mediterranea massiliensis*” Marseille-P2645 (GCA_900128475), and “*Bacteroides ndongoniae*” Marseille-P2644 (GCA_900128455) assigned as their closest relatives. However, none of these three species represents a validly named species, and thus in consequence, the first validly named phylogenetic relative to these two strains is Bacteroides clarus YIT 12056^T^ (GCA_000195615). Further phylogenomic analysis was performed by uploading genomes of strains AN20^T^, AN421^T^, and AN502 to the TYGS server, which compares query sequences with the type strain genomes in TYGS database using the MASH algorithm. Only the most closely related species were chosen for a pairwise comparison and calculation of the genome-based phylogenetic tree (GBDP). Strain AN20^T^ formed a distant lineage within a monophyletic cluster comprising *Phocaeicola salanitronis* DSM 18170^T^ and “*B. ndongoniae*” Marseille-P2644, with the latter not being a validly named species (Fig. S2, supplemental material). Strains AN421^T^ and AN502 clustered similarly as in MiGA analysis with nonvalidly named species “*B. togonis*” Marseille-P1366, “*Bacteroides mediterranensis*” Marseille-P1308, and “*Alistipes magaguti*” Marseille-P5997 and validly named Bacteroides faecichinchillae DSM 26883^T^ and Phocaeicola massiliensis DSM 17679^T^. TYGS server assigned all three strains as possible novel taxa within the family *Bacteroidaceae* for which the genera *Bacteroides* and *Phocaeicola* represent the closest related genera. As the TYGS server also includes type strains of nonvalidly named species, genomes of the valid species determined as the closest relatives by TYGS were subjected to CSI Phylogeny to infer an additional whole-genome-based phylogenomic tree. Similarly, as in the UBCG tree, three chicken cecum isolates formed a monophyletic clade within the family *Bacteroidaceae*, with clear separation of AN20^T^ from AN421^T^ and AN502 within the closest distance to a clade comprising six *Bacteroides* spp. and *C. zoogleoformans*, which clusters among *Bacteroides* spp., supporting its future reclassification (Fig. S3). Although classification of anaerobic bacteria is constantly evolving, phylogenomic analysis considering the most recent reclassifications clearly showed that strains AN20^T^, AN421^T^, and AN502 represent a distinct lineage within the family *Bacteroidaceae* and do not belong to any known genera from this family.

**FIG 3:**
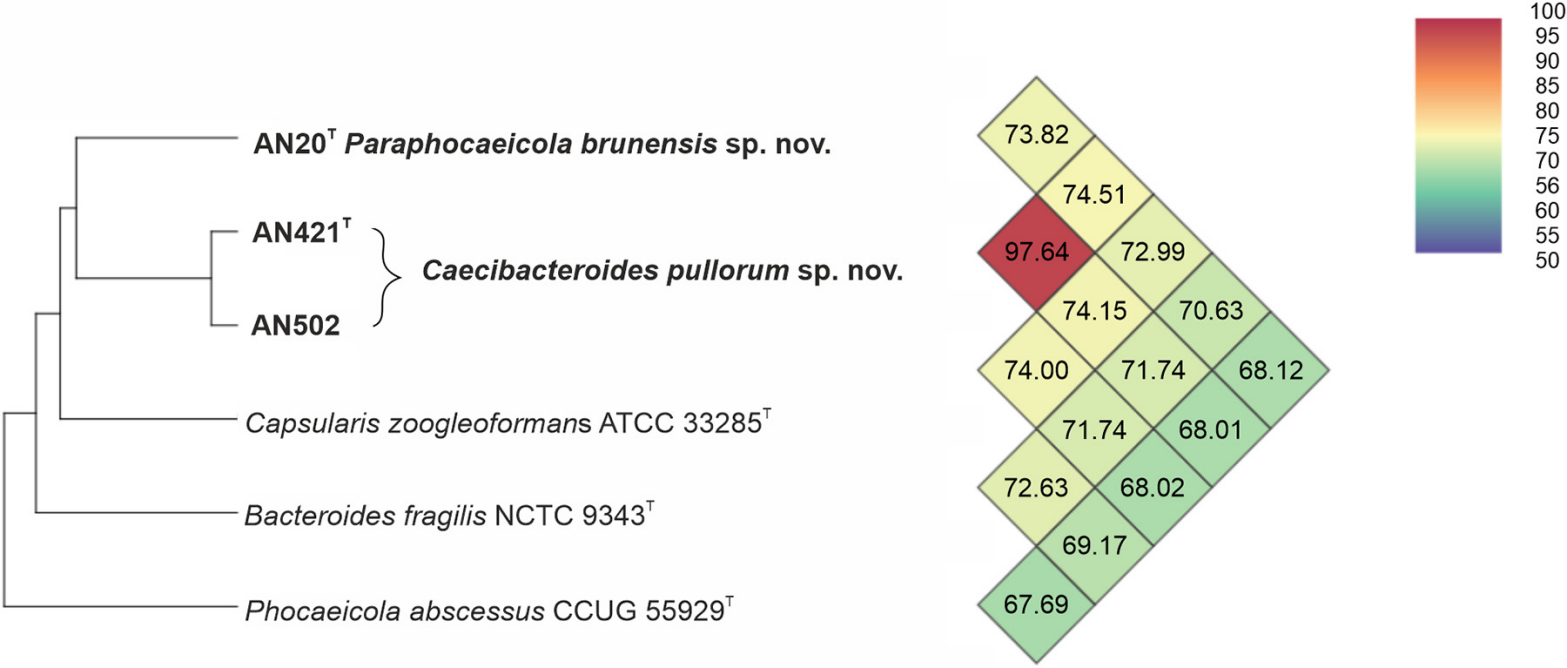
Heatmap generated with orthoANI values calculated using the OAT software (59).

The genomic similarities of AN20^T^, AN421^T^, and AN502 were further determined using calculation of genome-to-genome distance calculation (GGDC), orthologous average nucleotide identity (orthoANI), and percentage of conserved proteins (POCP) values. B. fragilis NCTC 9343^T^, *P. abscessus* CCUG 55929^T^, and *C. zoogleoformans* ATCC 33285^T^ were included as reference genomes in these calculations as they represent type species of related genera within the family *Bacteroidaceae*. The GGDC values between AN20^T^, AN421^T^, and AN502 and all three reference strains were all below 21.0% (Table 1). The GGDC values in between AN20^T^, AN421^T^, and AN502 were 22.5% and 25.2%, respectively. These values are far below the 70% cutoff value suggested for species delineation; however, no specific cutoff has been established for genera delineation so far (16). OrthoANI values in between AN20^T^, AN421^T^, AN502 and reference strains were all below 75%, with one exception, confirming that strains AN421^T^ and AN502 belong to one species (97.64%) (Fig. 3). Despite orthoANI not being suitable for precise species delineation (17), the results clearly showed that isolated strains do not belong to the aforementioned species or to any other closely related species of family *Bacteroidaceae*. A comprehensive table summarizing orthoANI values as well as 16S rRNA gene similarities between the closest phylogenetic neighbors is listed as Table S2 in the supplemental material. Qin et al. (17) suggested a novel taxonomic boundary for delineation of novel bacterial genera based on percentage of conserved proteins (POCP). Calculation of POCP values between AN20^T^ and B. fragilis, *P. abscessus*, and *C. zoogleoformans* showed 49.5%, 43.6%, and 50.5% of shared conserved proteins, respectively (Table 1). Strains AN421^T^ and AN502 shared with B. fragilis, *P. abscessus*, and *C. zoogleoformans* 50.3 to 50.5%, 47.7 to 45.7%, and 56.1 to 54.4% of conserved protein, respectively (Table 2). As suggested by Qin et al. (17), two taxa belong to the same genus if they share at least 50% of conserved proteins. Clearly, POCP values between the three cecum isolates and type species of related genera are in the range of this threshold, suggesting classification of these isolates to the genus *Capsularis*, although they do not phylogenetically cluster together. Similar discrepancies have already been observed within the families *Rhodobacteraceae*, *Bacillaceae*, and *Clostridiaceae*, where delineation of novel genera requires careful consideration of all genomic, phenotypic, and chemotaxonomic properties (17–19). By further example, Phocaeicola plebeius DSM 17135^T^ and *P. abscessus* CCUG 55929^T^ share 52% and 51.4% of conserved proteins with *C. zoogleoformans* ATCC 33285^T^, respectively, despite belonging to different genera, which suggests that POCP calculation may not be reliable for delineation of novel genera within the family *Bacteroidaceae*.

**TABLE 1:** GGDC values obtained from genomic comparison of AN20^T^, AN421^T^, AN502, and type species of closely related genera within family *Bacteroidaceae*

Strain	GGDC value for strain:					
	AN20^T^	AN421^T^	AN502	B. fragilis NCTC 9343^T^	*P. abscessus* CCUG 55929^T^	*C. zoogleoformans* ATCC 33285^T^
AN20^T^	100%					
AN421^T^	22.5%	100%				
AN502	25.2%	80.0%	100%			
B. fragilis NCTC 9343^T^	21.0%	20.9%	20.7%	100%		
*P. abscessus* CCUG 55929^T^	19.2%	19.0%	19.0%	19.3%	100%	
*C. zoogleoformans* ATCC 33285^T^	20.6%	21. 0%	21. 0%	21.2%	23.9%	100%

**TABLE 2:** POCP values for pair of genomes of strains AN20^T^, AN421^T^, AN502, and type species of closely related genera within family *Bacteroidaceae*

Strain	POCP value for strain:					
	AN20^T^	AN421^T^	AN502	B. fragilis NCTC 9343^T^	*P. abscessus* CCUG 55929^T^	*C. zoogleoformans* ATCC 33285^T^
AN20^T^	100%					
AN421^T^	55.7%	100%				
AN502	60.7%	80.8%	100%			
B. fragilis NCTC 9343^T^	49.5%	50.5%	50.3%	100%		
*P. abscessus* CCUG 55929^T^	43.6%	47.7%	45.8%	41.6%	100%	
*C. zoogleoformans* ATCC 33285^T^	50.5%	56.1%	54.4%	50.0%	51.4%	100%

### Genomic annotations.

**(i) Genome information and genomic properties of AN20^T^.** The draft genome of strain AN20^T^ was assembled into 88 contigs with the total genome size of 3,844,582 bp and the genomic G+C content of 49.52 mol%. The detailed draft genome characteristics are listed in Table S2. The draft genome was predicted to harbor a total of 3,445 genes out of which 3,294 represent protein coding genes (CDSs) by the Prokaryotic Genome Annotation Pipeline (PGAP). The genome contains 5 rRNA sequences (two copies of 5S and 23S rRNA and a single copy of 16S rRNA), 55 tRNAs, and 2 ncRNAs. Prophage Hunter detected 19 prophage regions, out of which 9 were assigned as active. A single CRISPR array was detected by CRISPR Detect with no *cas*-associated genes in close proximity. Out of a total, 2,180 genes accounting for 65.69% were assigned to a specific Cluster of Orthologous Genes (COGs) (Table S4). The remaining 1,114 genes (34.31%) were not assigned to specific COGs or their function remains unknown (category S). The majority of genes were assigned to category M, cell wall/membrane/envelope biogenesis, L, replication, recombination, and repair, E, amino acid transport and metabolism, and K, transcription.

**(ii) Genome information and genomic properties of AN421^T^.** The draft genome of strain AN421^T^ was assembled into 60 contigs with the total genomes size of 3,610,839 bp and the genomic G+C content of 48.66 mol%. The detailed draft genome characteristics are listed in Table S3. The draft genome was predicted to harbor a total of 3,008 genes, out of which 2,881 represent protein coding genes (CDSs) by PGAP. The genome contains 6 rRNA sequences (four copies of 5S and single copy each of 16S and 23S rRNA), 59 tRNAs, and 2 ncRNAs. Prophage hunter detected five prophage regions, out of which four were assigned as active. One reversely oriented CRISPR array from the II-C family was detected with a medium confidence associated with *cas9*, *cas2*, and *cas1* genes. Out of a total, 2,131 genes accounting for 73.91% were assigned to a specific cluster of orthologous genes (COGs) (Table S3). The remaining 752 genes (26.09%) were not assigned to specific COGs or their function remains unknown (category S). The majority of genes was assigned to category G, carbohydrate transport and metabolism, M, cell wall/membrane/envelope biogenesis, E, amino acid transport and metabolism, J, translation, ribosomal structure, and biogenesis, L, replication, recombination, and repair, C, energy production and conversion, K, transcription, and P, inorganic ion transport and metabolism.

**(iii) Genome information and genomic properties of AN502.** The draft genome of strain AN502 was assembled into 121 contigs with the total genomes size of 3,873,033 bp and the genomic G+C content of 48.40 mol%. The detailed draft genome characteristics are listed in Table S2. The draft genome was predicted to harbor a total of 3,256 genes out of which 3,129 represent protein coding genes (CDSs) by PGAP. The genome contains 4 rRNA sequences (two copies of 5S and a single copy each of 16S and 23S rRNA), 63 tRNAs, and 2 ncRNAs. Prophage hunter detected 16 prophage regions, out of which 4 were assigned as active. Four CRISPR arrays were detected with high confidence, out of which three were reversely oriented. Remaining forward-oriented CRISPR array consisting of nine repetitions was associated with *cas1*, *cas2*, and *cas9* genes. Out of a total, 2,226 genes accounting for 71.14% were assigned to a specific cluster of orthologous genes (COGs) (Table S3). The remaining 903 genes (28.86%) were not assigned to specific COGs or their function remains unknown (category S). The majority of genes was assigned to category M, cell wall/membrane/envelope biogenesis, G, carbohydrate transport and metabolism, L, replication, recombination and repair, E, amino acid transport and metabolism, and K, transcription.

### Genomic comparison.

**FIG 4:**
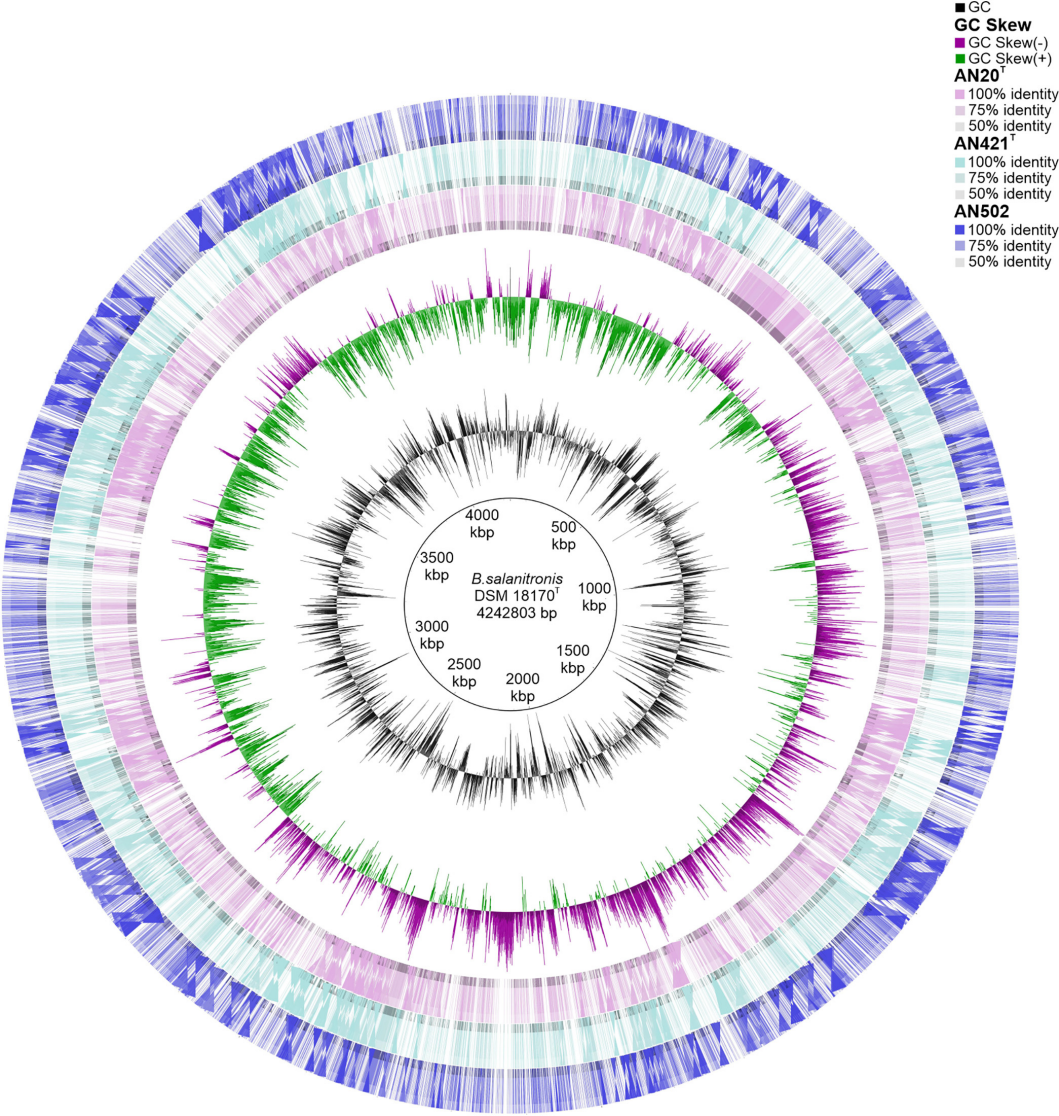
Circular genome representation of *Pseudophocaeicola brunensis* sp. nov. (AN20^T^) and *Caecibacteroides pullorum* sp. nov. (AN421^T^, AN502) with genome of *P. salanitronis* DSM 18170^T^. The innermost ring 1 represents the GC content, followed by ring 2 showing the GC skew (− and +); ring 3 is AN20^T^ (pink), ring 4 is AN421^T^ (turquoise), ring 5 is AN502 (blue), and ring 6 is *P. salanitronis* DSM 18170^T^ (red). Colors indicate the percentage of sequence identity.

Genomic comparison (Table S5) showed that the draft genomes of isolates AN20^T^, AN421^T^, and AN502 are of similar size, 3.84 Mb, 3.61 Mb, and 3.87 Mb, respectively. Circular maps of these three genomes and their comparison are shown in Fig. 4. These genomes are significantly smaller than B. fragilis NCTC 9343^T^ genome (5.24 Mb) and larger than *C. zoogleoformans* ATCC 33285^T^ and *P. abscessus* CCUG 55929^T^ with 3.36 and 2.54 Mb genome sizes, respectively. Significant difference lies in genomic G+C content where newly described chicken cecum isolates contain 49.5 (AN20^T^), 48.7 (AN421^T^), and 48.4 (AN502) mol% in comparison to *P. abscessus* CCUG 55929^T^ and *C. zoogleoformans* ATCC 33285^T^ with 47.2 mol% and 47.5 mol%, respectively, and significantly different B. fragilis NCTC 9343^T^ with the lowest genomic G+C content of 43.1 mol%. The genomic G+C content derived from whole-genome sequencing (WGS) data represents an important criterion of phylogeny relationships where intraspecies differences oscillate around 1% (20), supporting that none of these chicken isolates belongs to the compared species.

**FIG 5:**
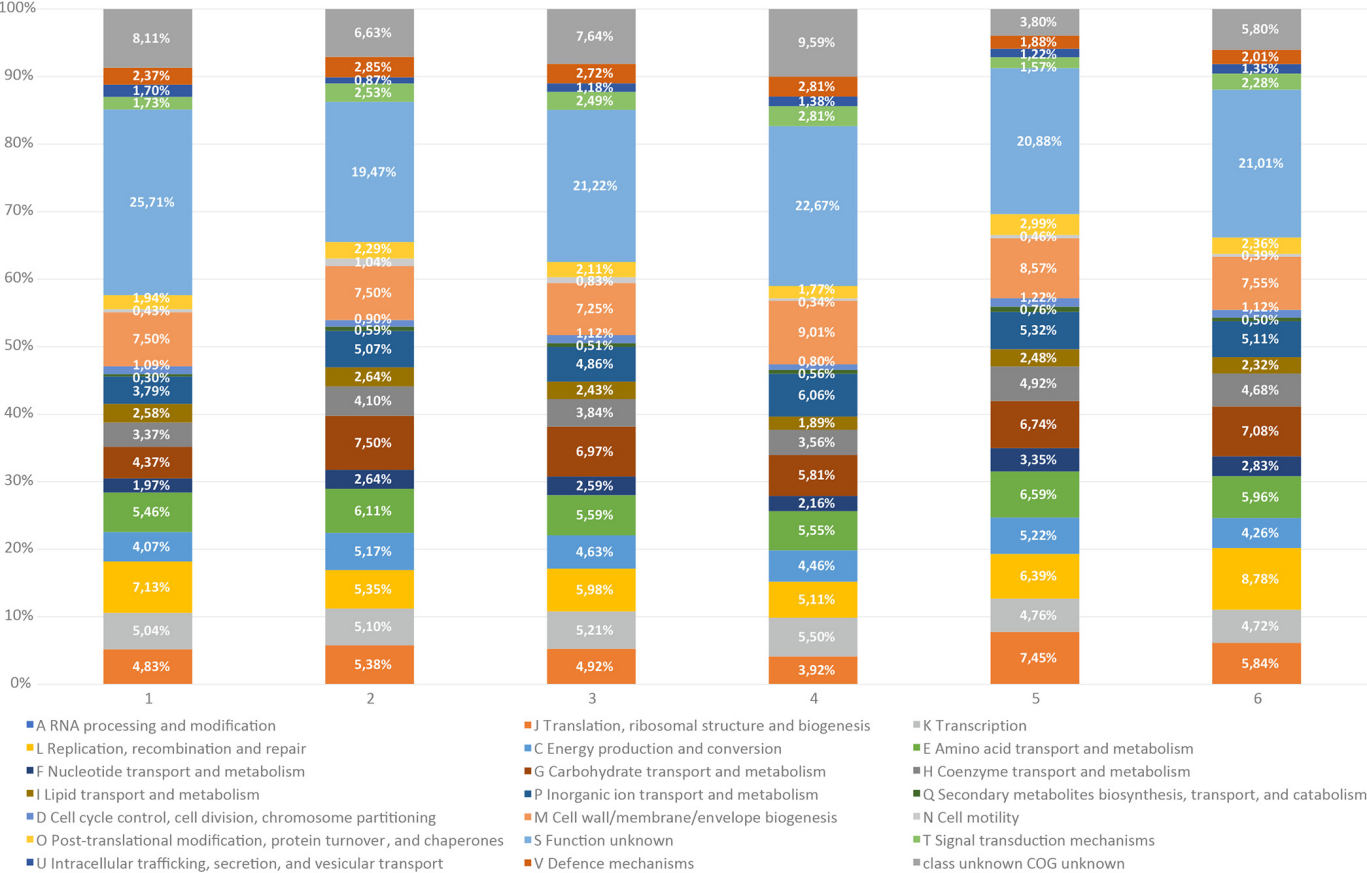
Comparison of COGs functional categories among isolated strains and the type species of the closely related genera within the family *Bacteroidaceae*. Strains: 1, AN20^T^; 2, AN421^T^; 3, AN502; 4, B. fragilis NCTC 9343^T^; 5, *P. abscessus* CCUG 55929^T^; 6, *C. zoogleoformans* CCUG 20495^T^. Each colored segment indicates the relative contribution of a functional category as a percentage of total COGs. The color of each COG family is indicated in the legend.

Comparison of functional annotation of AN20^T^, AN421^T^, and AN502 to reference strains showed significant differences in final gene contents (Table S4), which reflects variability observed in genome sizes. Comparing abundance of COGs assigned to specific categories, differences were found between two newly described genera as well as between them and their closest relatives (Fig. 5). All strains shared higher abundance of three functional categories (E, amino acid transport and metabolism, L, replication, recombination, and repair, and M, cell wall/membrane/envelope biogenesis). Strain AN20^T^ can be distinguished by lower proportion of genes assigned to categories J, translation, ribosomal structure, and biogenesis, and G, carbohydrate transport and metabolism, compared to that of all other analyzed strains. Strains AN402^T^ and AN502 have similar distributions of genes into COGs groups, differing from reference strains by quantitative differences, such as higher gene abundance in group J, translation, ribosomal structure, and biogenesis, than that of B. fragilis NCTC 9343^T^ and higher gene abundance in group K, transcription, than that of *P. abscessus* CCUG 55929^T^ and *C. zoogleoformans* ATCC 33285^T^.

### CRISPRs.

The CRISPR-Cas systems are represented by clustered regularly interspaced short palindromic repeats (CRISPR) and the CRISPR-associated (Cas) proteins. These systems act as an adaptive immune response of prokaryotes, providing protection against invading genetic elements and viruses (21). Three classes of CRISPR-Cas systems (I to III) are recognized among prokaryotes that fundamentally differ in organization and present nuclease (22). However, all CRISPR-Cas systems, regardless of the class or subsystem, act on three stages, with the first one involving acquisition of a foreign sequence followed integration as a novel spacer within the CRISPR array, followed by transcription of CRISPR array into pre-CRISPR RNA (pre-crRNA), which results in small crRNA molecules. During the final stage, DNA/RNA interference, small crRNA molecules guide effector Cas nuclease to cleave complementary sequence (21). CRISPR-Cas systems are associated with regulation of horizontal gene transfer (HGT), which is of particular interest for intestinal microbiota, as intestines represent a dense reservoir of antibiotic resistance genes and at the same time a “hot spot” of HGT between microbes (23). So far, only little is known about CRISPR-Cas systems among members of the family *Bacteroidaceae*, in particular about their function within gut microbiota and effect on HGT. The genus *Bacteroides* seems to be typical with the presence of CRISPR-Cas type II system, unlike the rest of the *Bacteroidetes* members (24). However, studies on B. fragilis isolates showed presence of three different CRISPR-Cas systems resembling type IB, type IIIB, and type IIC systems, which seem to be associated with different virulences of these strains (25). All three strains isolated in this study were found to contain CRISPR arrays, but only strains AN421^T^ and AN502 were found to harbor complete CRISPR loci with CRISPR array, type II-C, accompanied by a set of *cas* genes (*cas9*, *cas1*, and *cas2*) in close proximity. While strain AN421^T^ harbored one single CRISPR locus, four CRISPR arrays were detected within the genome of AN502, but three of them were not flanked by *cas* genes and may represent so-called orphans, putative isolated arrays (Table S6). However, these three arrays were located at the contig edges, they may belong to more complex arrays, and their separation is assembly bias associated with repetitive nature of these sequences (26). The fact that the isolated arrays in AN502 genome have the same sequence identity as the *cas*-adjacent CRISPR array 1 (Table S6) may, however, indicate an evolutionary relationship between these loci which may be functional, similar to isolated arrays found in Escherichia coli (27). Presence of CRISPR-systems facilitates studying and modifying organisms with unusual efficiency and has found its way into microbiome and probiotics research. So far, CRISPR engineering has already been employed for E. coli, lactobacilli, and clostridia aiming to improve growth rate, increase bile salt hydrolase activities, modify immunomodulatory properties, or suppress virulence (28, 29). Presence of CRISPR-systems is an important feature that supports a possible use of strains AN421^T^ and AN502 as future probiotics, which are already able to stably colonize chicken intestines and which could be easily modified with enhanced functionalities.

### Antibiotic resistance.

Two copies of *nimB* resistance genes were found in the AN20^T^ genome on scaffolds 7 and 12 with 95.35% and 100% gene sequence similarity to *nimB* gene of B. fragilis BF-8 (NG_048012.1) (Table S7). The *nimB* gene belongs to the group of genes currently comprising 11 recognized *nim* genes (*nim*A to *nim*K) encoding nitroimidazole-reductases, which are associated with acquired resistance to metronidazole, a 5-nitroimidazole antibiotic (30). *nim* genes were initially associated with diverse members of the phylum *Bacteroidetes* (*Bacteroides*, *Odoribacter*, *Parabacteroides*, *Porphyromonas*, *Prevotella*) and members of the B. fragilis group (31). These genes were subsequently demonstrated in a plethora of anaerobic bacteria, including genera such as *Clostridium*, *Veillonella*, *Fusobacterium*, *Cutinibacterium*, and *Actinomyces* (30, 31). Only about 50% of *nim* genes share a compatible G+C content with their hosts, suggesting that presence of these genes among anaerobic bacteria may result from a horizontal gene transfer (30). While some of these genes are located exclusively on plasmids, or plasmids and/or chromosomes, the *nimB* gene has been so far detected only on chromosomes (31, 32). Although *nimB* gene is located on chromosomes, it is usually associated with mobile genetic elements responsible for mobilization of various genes, including additional antibiotic resistance genes (ARGs) (33). *Bacteroides* spp. usually contain an insertion sequence located upstream of the *nimB* gene, typically IS1168 and less frequently IS612 and IS614 (32, 34). Interestingly, neither these nor other insertion sequence (IS) elements were found in close proximity to *nimB* genes found in AN20^T^ genome. The presence of *nimB* genes in AN20^T^ genome with sequence similarity of >95% to the *nimB* gene of B. fragilis suggests an interspecies horizontal gene transfer (5). This resistance transfer between *Bacteroides* spp. and other genera has already been established in human colon, and it is not surprising that a similar trend would be found in chicken intestines (7). Spread of *nim* genes in poultry is of concern, especially because nitroimidazoles are effective drugs for blackhead disease (histomoniasis) treatment in poultry, although they are currently prohibited for use in any food-producing animal species in the European Union by Council Regulation No. 2377/90 and Commission Regulation 2205/2001. Despite the fact that AN20^T^ contains two copies of *nimB* gene, it was found sensitive to metronidazole with MIC value of 0.064 mg/L (breakpoint 4 mg/L) with the ability to survive 3 mg/L after adaptation. It seems that *nimB* genes cannot be expressed in strain AN20^T^ or that these genes are simply silent in laboratory conditions, which is a known phenomenon described in association with *nimB*-positive yet metronidazole-sensitive strains of B. fragilis (30, 35).

Strain AN421^T^ harbored two antibiotic resistance genes, *lnuA_N2_* and *mefE_N2_* (Table S7), responsible for resistance to lincosamides and macrolides (36, 37). Both these genes were originally described from an mobilizable *Bacteroides* element, NBU2, found in clinical strains of B. fragilis ERL and Bacteroides thetaiotaomicron DOT (36). While resistance to lincosamides is conferred by O-nucleotidylyltransferase, which deactivates lincomycin and clindamycin, macrolides resistance is a result of an active MefA efflux pump complex (36). Both these genes were originally found as putative resistance genes sharing partial sequence similarities with ARGs of Gram-positive bacteria, *lnuA_N2_* 70 to 72% with *linA* gene of Staphylococcus aureus and Staphylococcus haemolyticus, respectively, and *mefE_N2_* 52 to 54% with *mefE* gene of Streptococcus pneumoniae and Streptococcus pyogenes, respectively (36). Expression studies showed that while *lnuA_N2_* provides the same resistance level as *linA* of Gram-positive bacteria, *mefE_N2_* does not seem to be expressed even when multiple copies of genes are introduced to the genome (36). Interestingly, antibiotic susceptibility testing showed that the strain AN421^T^ was resistant to erythromycin (MIC > 256 mg/L) while sensitive to lincosamides (MIC of clindamycin 0.50 mg/L). Strain AN502, which was not found to contain any resistance genes, was found sensitive to both erythromycin (MIC 2.0 mg/L) and clindamycin (MIC < 0.002 mg/L).

### Ecological role and host specificity of the analyzed chicken cecum isolates.

Any host-associated microbiomes represent sophisticated communities with complex relationships to their hosts. The three chicken isolates described within this work were initially isolated from newly hatched chickens, which is valuable for possible use of these strains as probiotics. In other to define host specificity regarding analyzed strains, distribution of these strains was investigated across different microbiomes of warm-blooded animals and humans. Using a BLAST comparison tool, 16S rRNA sequence of each strain was queried against three subsets of metagenomes present in the JGI IMG/M database, human-, mammal (excluding human metagenomes)-, and bird-associated, to find presence of related 16S rRNA sequences. NCBI BLAST tool was further used to find any isolates or uncultured bacteria related to analyzed strains within the GenBank database. For AN20^T^, the closest reference match within the JGI IMG/M metagenomic database was a short sequence (731 bp) of 99% similarity present in a chicken fecal sample from China (JGI ID: 3300029905). Search against the NCBI database showed four almost-complete 16S rRNA gene sequences of uncultured bacteria (99.71 to 99.11%) found in preadolescent turkeys as the closest match. For strains AN421^T^ and AN502, the highest matching 16S rRNA sequence (100%) was found in metagenomic data analyzed in Denmark from fecal microbial communities from an infant at 12 months (JGI ID: 3300029180). This could indicate that strains AN421^T^ and AN502 are associated primarily with juvenile hosts. However, blast search against the GenBank database showed that the closest sequence matches (≥98.65%) for strain AN421^T^ (99.64 to 98.75%) and strain AN502 (99.93 to 98.69%) were also represented by a group of 29 and 31 uncultured bacteria, respectively, found in fecal human communities and human iliac mucosa-associated microorganisms. Presence of these almost-complete 16S rRNA sequences with sequence identities higher than the species delineation threshold in fecal and iliac samples suggests that members of this species are able to colonize not only juvenile poultry and human infants but also human adults. On the contrary, strain AN20^T^ seems to be associated according to available data only with avian gastrointestinal tracts. It is also important to notice that prevalence of related 16S rRNA gene sequences is far lower than that of *Bacteroides* or *Phocaeicola* species. All sequences from uncultured clones (Table S8) were obtained from only five different studies (two avian-oriented, three human-oriented), which represents a small fraction compared to the number of genera *Bacteroides* and *Phocaeicola* sequences (474,543 and 98,560 nucleotide sequences present in the GenBank database, respectively). Additive studies are required to estimate prevalence of the two novel genera in human or animal intestinal tracts, which will be facilitated and more efficiently communicated after official classification of these genera.

### Phenotypic characteristics.

Colonies on supplemented Wilkins-Chalgren anaerobe agar (WCHA) agar following cultivation for 2 days were white or grayish white, circular, entire, low convex, and 1.0 to 1.7 mm in diameter. Hemolytic activity was observed on the Columbia blood agar plates after 2 and 4 days of cultivation. Cells were non-spore-forming, nonmotile, Gram-stain-negative short to longer rods, and filamentous structures were also observed (Fig. S4 and S5). All three isolates were obligately anaerobic. Optimal growth of all strains was observed at 37°C and pH near 7. Rumen fluid extract supplementation stimulated growth of all strains. Growth on WCHA agar without supplementation was significantly weaker than growth with supplementation. The pH range of all three isolates was 6.0 to 10.0. Concentration of 2.0% NaCl inhibited growth of AN20^T^ and 1.5% NaCl inhibited growth of AN421^T^ and AN502. AN20^T^ hydrolyzed gelatin and weakly hydrolyzed cellulose, whereas AN421^T^ and AN502 were positive for gelatin hydrolysis only. Strain AN20^T^ was resistant to bile acids. The major end products of glucose metabolism of AN20^T^ were acetate (54.7 mM) and succinate (25.4 mM), whereas for AN421^T^ and AN502, only acetate was found in larger amounts (51.3 and 51.4 mM, respectively). Formate, malate, and butyrate were produced in minor amounts by all strains. Lactate was produced only by AN421^T^ and AN502. All three strains were negative for propionate production. Although data for fermentation end products typical for the genus *Phocaeicola* are not available, as this genus comprises mainly former *Bacteroides* spp., it is likely that these two genera have similar fermentation patterns, comprising mainly acetate and succinate as the major end products from glucose fermentation and butyrate, isobutyrate, isovalerate, propionate, phenylacetate, formate, and lactate as minor products (38). Members of the genus *Prevotella* are characterized by a similar pattern, producing acetate and succinate as the major products from glucose fermentation and short-chain fatty acids in minor amounts (39). While the major end products of glucose metabolism of AN20^T^ are similar to those of the closely related genera, strains AN421^T^ and AN502 differ by producing predominantly acetate. The results of phenotyping based on API RAPID ID 20A and 32A systems are specified in the species description listed below. Several biochemical tests that can be used for distinguishing members of both groups from closely related genera within the family *Bacteroidaceae* and from each other are listed in Table 3. A more comprehensive table including all tested phenotypic characteristics is included in the supplemental material (Table S9).

**TABLE 3:** Phenotypic tests differentiating AN20^T^, AN421^T^, AN502, and the type species of the closely related genera within the family *Bacteroidaceae*

Test or characteristic	Result for strain:^*a*^					
	*Pseudophocaeicola brunensis* sp. nov. AN20^T^	*Caecibacteroides pullorum* sp. nov.		B. fragilis CCM 4712^T^	*P. abscessus* DSM 21584^T^	*C. zoogleoformans* CCUG 20495^T^
		AN421^T^	AN502			
Cell size	0.8–0.9 by 1.8–20 μm	0.4–1.2 by 0.6–24 μm	0.4–1.2 by 0.6–24 μm	0.8–1.3 by 1.6–8.0 μm^*b*^	0.3–0.6 by 0.4-0.9 μm^*c*^	0.6–1.0 by 0.8–8.0 μm^*d*^
Temp range	28–47°C	30–40°C	28–47°C	25–45°C	30–37°C	25–37°C
pH range	6.0–12.0	6.0–11.0	6.0–12.0	5.0–12.0	6.0–7.0	7.0–12.0
NaCl tolerance	0.0–1.0%	0.0–0.5%	0.0–0.5%	0.0–2.0%	0.0–0.5%	0.0–1.0%
Catalase	−	−	−	+	−	−
Resistance to bile acids	+	−	−	+	−	−
Hydrolysis of:						
Gelatin	+	+	+	−	−	−
Esculin	−	−	−	+	−	+
Cellulose	w	−	−	w	−	−
RAPID 20A						
GLU	w	+	+	+	−	−
LAC	+	+	+	+	w	−
SAC	−	+	+	+	−	−
MAL	+	+	+	+	−	−
XYL	−	+	−	+	−	−
ARA	−	+	−	−	−	−
GEL	+	+	+	+	−	w
ESC	−	−	−	+	−	−
MNE	+	+	+	+	−	−
RAF	−	+	+	+	w	−
RHA	−	+	−	−	−	−
API RAPID 32A						
αGAL	+	+	+	−	+	−
βGAL	+	+	+	+	+	+
αGLU	−	+	+	+	−	−
MNE	−	+	+	−	−	−
GDC	−	−	−	+	+	−
αFUC	+	+	+	w	+	−
ArgA	−	+	+	−	−	−
PheA	−	+	+	−	−	−
LeuA	−	+	+	−	−	−
GGA	−	−	−	w	−	−
SerA	−	−	−	−	−	−

a +, positive result; −, negative result; w, weak reaction.

b Data were taken from (38).

c https://bacdive.dsmz.de/.

d Data were taken from (39).

### Chemotaxonomic characteristics.

The predominant fatty acids (>10%) for all analyzed strains were iso-C_15:0_, anteiso-C_15:0_, C_16:0_, C_18:1_ *ω9c*, iso-C_17:0_ 3OH, and additionally, C_16:0_ 3OH for AN421^T^ and AN502. Comparison to reference type strains showed similar fatty acid profiles with mostly quantitative differences that separate strains AN20^T^, AN421^T^, and AN502 from closely related genera and from each other (Table 4). Major differences are in larger amounts of anteiso-C_15:0_ and iso-C_17:0_ 3OH in B. fragilis CCM 4712^T^, larger amounts of iso-C_13:0_ and iso-C_15:0_ in *C. zoogleoformans* CCUG 20495^T^, and significantly smaller amount of iso-C_15:0_ in *P. abscessus* DSM 21584^T^ compared to those in the chicken cecum isolates. Differences between the two proposed genera are similarly quantitative, relating mainly to amounts of medium-chain fatty acids iso-C_13:0_ and C_14:0_, branched fatty acid iso-C_17:0_, and hydroxy fatty acids C_16:0_ 3OH and C_17:0_ 2OH. The major menaquinones of AN20^T^ were MK-5 (60.8%) and MK-11 (29.32%), while the major menaquinones of AN421^T^ and AN502 were MK-5 (58.5% and 55.1%, respectively) and MK-10 (35.5% and 40.1%, respectively). AN421^T^ and AN502 contained minor amounts of MK-11, whereas AN20^T^ contained minor amounts of MK-12. Specific combinations of menaquinones in isolated strains represent a significant chemotaxonomic feature clearly separating isolate AN20^T^ from AN421^T^ and AN502 as well as both these lineages from their closest relatives. The genus *Bacteroides* is characteristic with the major menaquinones comprising MK-10 and/or MK-11 (38). Menaquinone spectrum of the genus *Phocaeicola* is not yet defined; however, this genus was recently amended and as a result now comprises mainly former *Bacteroides* spp. (2), with a majority of them containing MK-10, MK-11, and MK-12 as major respiratory quinones, with the exception of MK-9 in Phocaeicola sartorii (38, 40–43). Unfortunately, information on respiratory quinones characteristic for the genus *Capsularis* and the type species *C. zoogleoformans* is lacking, although this information may have additive value for taxonomic resolutions regarding this genus. Nonetheless, two genera, *Phocaeicola* and *Bacteroides*, are distinguishable from both newly described genera originating from chicken ceca for which the presence of the major respiratory quinone MK-5 appears to be a specific characteristic.

**TABLE 4:** Cellular fatty acid composition (as a percentage of the total) of strains AN20^T^, AN421^T^, AN502, and type species of the closely related genera^*a*^

Fatty acid	Value for strain:					
	*Paraphocaeicola brunensis* sp. nov. AN20^T^	*Caecibacteroides pullorum* sp. nov.		B. fragilis CCM 4712^T^	*P. abscessus* DSM 21584^T^	*C. zoogleoformans* CCUG 20495^T^
		AN421^T^	AN502			
iso-C_13:0_	TR	ND	ND	TR	ND	3.0
C_14:0_	4.2^*b*^	2.2	2.0	1.2	TR	4.9
iso-C_15:0_	12.0	8.5	15.1	10.3	3.7	21.6
anteiso-C_15:0_	12.0	8.9	8.8	19.4	14.6	12.1
C_16:0_	9.7	10.7	6.7	8.6	10.7	9.8
iso-C_17:0_	3.8	TR	TR	2.3	1.1	TR
anteiso-C_17:0_	1.2	1.3	1.0	1.7	3.0	TR
C_16:0_ 3OH	6.1	16.0	8.5	5.2	6.1	5.7
C_18:1_ *ω9c*	23.6	25.6	24.7	18.1	26.5	19.9
iso-C _17:0_ 3OH	13.3	11.0	18.5	30.0	19.6	10.1
C _17:0_ 2OH	TR	1.8	1.3	2.0	2.9	TR
C _17:0_ 3OH	TR	1.3	TR	TR	1.2	ND
Summed feature 3^*c*^	2.4	2.6	2.6	2.0	2.9	3.7
Summed feature 8	4.2	4.4	4.3	2.7	3.9	3.0

a ND, not determined; TR, trace amounts. Values of less than 1% are not shown. All data were obtained in this study.

b The values listed are expressed as means of two duplicates.

c Summed features represent groups of fatty acids that cannot be separated by gas chromatography using the MIDI system. Summed feature 3, C_16:1_ *ω7c*/C_16:1_ *ω6c*; summed feature 8, C_18:1_ *ω7c*/C_18:1_ *ω6c*.

### Conclusion (includes descriptions).

In this study, three novel anaerobic strains (AN20^T^, AN421^T^, and AN502) were isolated from chicken ceca. Analyses of 16S rRNA genes, core genes, and whole-genome phylogenetics showed that these strains form a separate lineage within the family *Bacteroidaceae*. Genomic relatedness was evaluated using ANI, POCP, and GGDC values and showed distinctiveness of analyzed strains from their phylogenetic relatives. Further phenotypic and chemotaxonomic results confirmed that analyzed strains differ from the phylogenetically closest genera *Bacteroides*, *Phocaeicola*, and *Capsularis* (Table 3). Compared to each other, genomic characteristics, as well as chemotaxonomy and phenotype, clearly distinguish strain AN20^T^ from both AN421^T^ and AN502, which form a separate phylogenetic line. Thus, these three isolates represent two novel genera belonging to the family *Bacteroidaceae*, and their descriptions are included below.

**Description of *Paraphocaeicola* gen. nov.** *Paraphocaeicola* (Pa′ra.pho.cae.i.co′la. Gr. prep. *para* beside; N. L. masc. n. *Phocaeicola* a genus name; N. L. masc. n. *Paraphocaeicola* resembling the genus *Phocaeicola*). Cells are anaerobic, Gram-negative, nonmotile rods. Catalase and oxidase activities are negative.

**Description of *Paraphocaeicola brunensis* sp. nov.** *Pseudophocaeicola brunensis* (bru.nen′sis. L. adj. *brunensis* from *Bruna*, the Roman name of the city of Brno, Czech Republic, where the type strain was isolated).

The cells are strictly anaerobic, non-spore-forming, nonmotile, Gram-stain-negative rods, short or longer rod shaped, usually 0.8 to 0.9 μm in width and variable in length, in the range 1.8 to 20 μm. Colonies on WCHA agar plates after 2 days are grayish white, circular, entire, low convex, 1.5 to 1.7 mm in diameter. Hemolytic activity was observed on the Columbia blood agar plates after 2 days of cultivation. Does not require NaCl for growth, 2.0% (wt/vol) NaCl inhibits growth. Cells grow at 28 to 47°C and pH 6.0 to 12.0. Growth is not inhibited on medium containing 20% (wt/vol) bile. Aesculin is not hydrolyzed in esculin agar. Indole is not produced. Catalase, oxidase, and urease are negative. Nitrates are not reduced. Gelatin is liquified. No activity was observed on medium with carboxymethylcellulose (CMC). Does weakly utilize cellulose as a sole carbon source. Positive for d-glucose (weakly), d-lactose, d-maltose, gelatin, and d-mannose in API 20A. Negative for indole, urease, mannitol, d-saccharose, salicin, d-xylose, l-arabinose, esculin, glycerol, d-cellobiose, d-melezitose, d-raffinose, d-sorbitol, l-rhamnose, and d-trehalose in API 20A. Using the API Rapid ID 32A, positive reactions were obtained for α-galactosidase, β-galactosidase, N-acetyl-β-glucosaminidase, α-fucosidase, alkaline phosphatase, leucyl glycine arylamidase, and alanine arylamidase. Negative reactions are obtained for urease, arginine dihydrolase, β-galactosidase-6-phosphate, α-glucosidase, β-glucosidase, α-arabinosidase, β-glucuronidase, d-mannose and d-raffinose fermentation, glutamic acid decarboxylase, reduction of nitrates, production of indole, arginine arylamidase, proline arylamidase, phenylalanine arylamidase, leucine arylamidase, pyroglutamic acid arylamidase, tyrosine arylamidase, glycine arylamidase, histidine arylamidase, glutamyl glutamic acid arylamidase, and serine arylamidase. The major end products of glucose fermentation were succinate and acetate with a smaller amount of formate and lactate and minor amount of butyrate. Does not produce lactate and propionate. The major fatty acids are C_18:1_ *ω9c*, iso-C_17:0_ 3OH, iso-C_15:0_, anteiso-C_15:0_, and C_16:0_. The major respiratory quinones are MK-5 and MK-11. Menaquinones MK-10 and MK-12 are also present as minor components. The DNA G+C content of the type strain is 49.52 mol% and genome size 3.84 Mb.

Type strain AN20^T^ (= CCM 9041^T^ = DSM 111154^T^) was isolated from the cecum of a chicken in Brno, Czech Republic.

**Description of *Caecibacteroides* gen. nov.** *Caecibacteroides* (Cae.ci.bac.te.ro′i.des. Gr. prep. caeci from N.L. neut. n. *caecum* cecum; N.L. masc. n. *bacter*, rod; L. adj. suff. -*oides*, resembling, similar; N.L. masc. n. *Caecibacteroides*, rods isolated from the cecum). Cells are anaerobic, Gram-negative, non-spore-forming, and nonmotile rods. Catalase and oxidase activities are negative.

**Description of *Caecibacteroides pullorum* sp. nov.** *Caecibacteroides pullorum* (pul.lo’rum. L. gen. pl. n. *pullorum*, chicken; referring to the source of isolation).

The cells are strictly anaerobic, non-spore-forming, nonmotile, Gram-stain-negative rods, short or longer rod shaped, 0.4 to 1.2 μm in width, and variable in length, in the range 0.6 to 24 μm. Colonies on WCHA agar plates after 2 days are grayish white, circular, entire, low convex, 1.0 mm in diameter. Hemolytic activity was observed on the Columbia blood agar plates after 4 days of cultivation. Does not require NaCl for growth, 1.0% (wt/vol) NaCl inhibits growth. Cells grow at 30 to 40°C and pH 6.0 to 11.0. Growth is inhibited on medium containing 20% (wt/vol) bile. Aesculin is not hydrolyzed in esculin agar. Indole is not produced. Catalase, oxidase, and urease are negative. Gelatin is liquified. No growth is observed on medium with CMC. Does not utilize cellulose as a sole carbon source. Positive for d-glucose, d-lactose, d-saccharose, d-maltose, gelatin, d-mannose, and d-raffinose utilization in API 20A. Negative for indole, urease, mannitol, salicin, esculin, glycerol, d-cellobiose, d-melezitose, d-sorbitol, and d-trehalose in API 20A. Using the API Rapid ID 32A, positive reactions were obtained for α-galactosidase, β-galactosidase, α-glucosidase, N-acetyl-β-glucosaminidase, d-mannose fermentation, α-fucosidase, alkaline phosphatase, arginine arylamidase, leucyl glycine arylamidase, phenylalanine arylamidase, leucine arylamidase, and alanine arylamidase. Negative reactions are obtained for urease, arginine dihydrolase, β-galactosidase-6-phosphate, β-glucosidase, α-arabinosidase, β-glucuronidase, d-raffinose fermentation, glutamic acid decarboxylase, reduction of nitrates, production of indole, proline arylamidase, pyroglutamic acid arylamidase, tyrosine arylamidase, glycine arylamidase, histidine arylamidase, glutamyl glutamic acid arylamidase, and serine arylamidase. The major end product of glucose fermentation is acetate with a smaller amount of lactate, succinate, and formate and minor amount of butyrate. Does not produce propionate. The major fatty acids are C_18:1_ *ω9c*, iso-C_17:0_ 3OH, C_16:0_ 3OH, iso-C_15:0_, anteiso-C_15:0_, and C_16:0_. The major respiratory quinones are MK-5 and MK-10. Menaquinone MK-11 was also present as minor component. The DNA G+C content is 48.35 to 48.66 mol% (type strain, 48.66 mol%) and genome size is 3.74 Mb.

Type strain AN421^T^ (= CCM 9040^T^ = DSM 111155^T^) was isolated from the cecum of a chicken in Brno, Czech Republic. Strain variable tests are temperature range, reduction of nitrates, and acidification of d-xylose, l-arabinose, and l-rhamnose in API 20A.

## MATERIALS AND METHODS

### Enrichment and isolation.

Pure cultures of three strains, assigned as AN20^T^, AN421^T^, and AN502, used in this follow-up study were originally isolated from chickens (Lohmann Brown Light variety) within the project focused on functional description of chicken gut microbiota published by Medvecky et al. (10). Isolates were cultivated on the same agar as used in the original study, Wilkins-Chalgren anaerobe agar (WCHA) (Oxoid) supplemented with 30% of rumen fluid, 10 mL/L hemin, 1.0 g/L cellobiose, 1.0 g/L soluble starch, 1.0 g/L maltose, 0.2 mL/L vitamin K1 solution (0.1 mL of filter sterilized vitamin K1 in 20 mL 95% ethanol), and 0.5 g/mL l-cysteine. The rumen fluid was collected from cows by an oral probe as described by Medvecky et al. (10). Sensitivity of pure anaerobe cultures to air oxygen exposure was tested as described by Browne et al. (44). Medium for long-term storage in −80°C contained prereduced anaerobically sterilized (PRAS) dilution with 20% glycerol and equal volume of sterile sheep blood. Unless mentioned otherwise, the isolates in this study were cultured on supplemented WCHA agar as mentioned above.

### Morphology.

Colony morphology was assessed on WCHA agar supplemented with rumen fluid as described above and on Columbia blood agar (Oxoid) supplemented with 5% sheep blood. Cell morphology was characterized by light microscopy (Leica DM 2000 LED, Switzerland) following Gram-staining and by scanning electron microscopy (SEM) using a Tescan Vega scanning electron microscope (Czech Republic). Samples for SEM were prepared from pure cultures by fixing the cells in 3% glutaraldehyde in 0.1 M phosphate buffer (pH 7.2) for 1 h. Cells were washed three times in buffer and passed through SPI-pore polycarbonate track filters (SPI Supplies, West Chester, PA, USA) held inside a 13-mm Swinnex filter unit (Millipore, Billerica, MA, USA), followed by dehydrating in 30-50-75-90-100-100% ethanol. Sample filters from 100% ethanol were dried for an hour, followed by mounting the filter onto SEM stub, and sputter-coated (10 nm gold/palladium) before imagining (45).

### Phylogenetic, phylogenomic, and genomic analyses.

The DNA was extracted by NucleoSpin Tissue Genomic DNA kit following the instructions of the manufacturer (Macherey-Nagel). Briefly, 10^7^ cultured bacterial cells were prelysed with 180 μL buffer T1 and 25 μL proteinase K at 56°C. After 3 h of incubation, 200 μL of buffer B3 was added to lyse the samples and incubated at 70°C/10 min. DNA binding conditions were adjusted with 210 μL ethanol (96%), and samples were loaded into the column and centrifuged at 11,000 × *g* for 1 min. Silica membrane was washed using 500 μL BW buffer and 600 μL B5 buffer. DNA was eluted to the 50 μL of elution buffer.

For phylogenetic analysis, the almost-complete 16S rRNA genes of all strains were amplified using universal primers EU16SrRNA/F (5′-AGAGTTTGATCITGGCTCAG-3′) and EU16SrRNA/R (5′-ACGGITACCTTGTTACGACTT-3′) (46). The initial denaturation was for 3 min at 94°C, followed by 30 cycles of denaturation at 90°C for 50 s, annealing at 50°C for 50 s, and elongation at 72°C for 2 min. The final extension step was for 10 min at 72°C. A single DNA band with length of ∼1,500 bp was observed in all strains on 1.5 % agarose gel stained with 2% Midori green advance dye (Nippo Genetics). Amplicons were purified using NucleoSpin gel and PCR clean-up kit according to manufacturer’s instructions (Macherey-Nagel) and sequenced using a commercially available service (Macrogen-Europe sequencing service).

The whole-genome sequencing was performed as described by Medvecky et al. (10). Briefly, Nextera XT DNA sample preparation kit (Illumina) was used for preparation of the sequencing library, and the NextSeq 500/550 high output kit v2 and Illumina NextSeq 500 sequencing platform were used for the whole-genome sequencing. Trimmomatic v0.32 (47) was applied for quality trimming of the raw sequencing reads, and the trimmed-paired reads were assembled *de novo* using IDBA-UD v1.1.1 (48). SSPACE scaffolder v3.0 (49, 50) was used to scaffold only high-coverage contigs longer than 500 bp.

The similarities of the 16S rRNA sequences between the three isolates and their closest phylogenetic neighbors were determined using EzBioCloud database (12). Phylogenetic analyses were inferred by both maximum-likelihood (ML) and Bayesian inferences (BI) using the 16S rRNA sequences of the closest validly named *Bacteroides* spp., all validly named *Phocaeicola* spp., and Capsularis zoogleoformans retrieved from the GenBank database. Fibrobacter succinogenes ATCC 19169^T^ was used as an outgroup to root the trees. All 16S rRNA gene sequences were aligned using CLUSTAL W algorithm in MEGA v7.0 software (51), genetic distances were calculated by Kimura’s two-parameter model, and the evolutionary history was inferred using the ML method. Confidence levels of observed clades were evaluated by bootstrap values based on 1,000 replicates. BI analysis was performed using BEAST2 (v2.6.6) (52) using Markov chain Monte Carlo (MCMC) with the general time reversible (GTR) nucleotide substitution model with a gamma (Γ)-distributed rate variation across sites in a relaxed clock log normal analysis (i.e., the GTR plus Γ substitution model). MCMC chains were run for 1,000,000 generations, sampling every 1,000th tree. Among these, the first 10% of trees were discarded as burn-in phase and the remaining trees were used to calculate Bayesian posterior probabilities. Quality of the MCMC output was evaluated using Tracer (v1.7.7.) software package (53), and final tree was visualized using FigTree v1.4.4. (https://github.com/rambaut/figtree/).

Draft or complete genome sequences of all validly described species (Table S1) identified as the closest phylogenetic neighbors were downloaded from the NCBI database (https://www.ncbi.nlm.nih.gov/). These genomes along with draft genomes of the three analyzed strains were subjected to the UBCG software and pipeline to generate phylogenetic trees from sequences of 92 concatenated core genes (15). Core gene set from Parabacteroides johnsonii DSM 18315^T^ (GCA_000156495.1) was used as outgroup to root the tree. To further refine taxonomic position of the three isolates based on whole-genome data, the draft genome sequences were submitted to the Microbial Genome atlas (MiGA) webserver (54) and to the type strain genome server (TYGS) (55) for additional analyses on phylogenomic classifications. TYGS server compares queried sequences with the type strain genomes in TYGS database using the MASH algorithm (56). The closest related species were then chosen for pairwise comparison and calculation of the genome-based phylogenetic tree (GBDP) and intergenomic distances were used to calculate minimum evolution phylogeny using FastME 2.1.6.1 (57). A further genome-based phylogenomic tree was inferred using the CSI Phylogeny v1.4 (58) accessible from the Center for Genomic Epidemiology (www.genomicepidemiology.org) and with Bacteroides fragilis NCTC 9343^T^ (GCA_000025985.1) used as a reference. Genome distances were further estimated by calculation of average nucleotide identities (orthoANI) using OAT software (59), digital DNA-DNA hybridization values using the genome-to-genome distance calculation (GGDC) (16), and percentage of conserved proteins (POCP) (17) using a ruby script (https://github.com/hoelzer/pocp).

Gene predictions and functional annotations were performed by Prokaryotic Genome Annotation Pipeline (60, 61) and EggNOG v5.0 (62) annotation tool. Presence of RNA genes was checked by BAsic rapid rRNA predictor (Barrnap v0.9, https://github.com/tseemann/barrnap). A visual genome comparison between AN20^T^, AN421^T^, and AN502 and reference strain *Phocaeicola salanitronis* DSM 18170^T^ at the nucleotide level was generated using the BLAST Ring Image Generator (BRIG) (63). Identification of the antimicrobial resistance genes, putative virulence factors, and plasmid-associated genes was performed using ABRICATE v1.0.1 (GNU, Boston, MA, USA; https://github.com/tseemann/abricate) and the following databases, respectively: Comprehensive Antibiotic Resistance Database (CARD) (64), ResFinder v. 3.2 (65) and MEGARES v2.00 (66), ARG-ANNOT (67) and NCBI (68), and PlasmidFinder 2.1 (69) and the virulence factor database (VFDB) (70). Insertion sequences, transposons, CRISPRs, and phages were identified using ISfinder (71), CRISPR Detect (72), Prophage Hunter (73), and Phaster (74). All data were accessed between 1 July 2021 and 20 July 2021.

### Physiological and biochemical analyses.

Physiological and biochemical tests were performed in triplicates for all three analyzed strains. Temperature range and NaCl tolerance were determined based on growth on supplemented WCHA agar (as described above) because the growth on WCHA agar without rumen fluid supplement was significantly weaker for all three analyzed strains. The pH tolerance was determined on the same agar with pH aseptically adjusted after autoclaving. Inoculated plates were incubated anaerobically for a maximum of 7 days at 37°C. Growth was tested at different temperatures ranging from 25 to 55°C in 5°C increments. Salt tolerance was assessed at 0.0 to 2.5% (at 0.5% increments) and pH tolerance at pH 5 to 13 at intervals of 0.5 pH unit by using the following buffer systems: pH 5.0 to 8.0, 0.1 M KH_2_PO_4_/0.1 M NaOH (pH 9.0 to 10.0), 0.1 MNaHCO_3_/0.1 M Na_2_CO_3_. Aerobic growth was assessed using the same agar medium as for above-mentioned growth tests. Catalase was determined using 3.0% (vol/vol) hydrogen peroxide solutions (75) and oxidase with diagnostic strips according to manufacturer’s instructions (OXItest; Erba Lachema). Hydrolysis of gelatin was performed on WCHA agar plates supplemented as described above with addition of 0.4% gelatin (pH 7 to 7.5, sterilization 115°C/20 min). Clear zones of gelatin hydrolysis were read after using Frazier’s reagent (76). Aesculin hydrolysis was determined on bile-esculin agar (Oxoid) and esculin agar (77), both supplemented with 10 mL/L hemin and 0.2 mL/L vitamin K1 solution after autoclaving. Hydrolysis of carboxymethylcellulose (CMC) and cellulose was determined on cellulose agar (78) supplemented with CMC and strip of Whatman Paper N.1, respectively. Bile resistance was determined using WCHA agar plates (as described above) supplemented with 20% (vol/wt) bile acids after autoclaving. Urease activity was detected according to the method of Barry et al. (79). Tests for indole production and nitrate reduction were prepared according to rapid protocols for testing of anaerobic bacteria (80). Further biochemical properties and enzymatic activities were tested in triplicates using API RAPID 20A and 32A strip tests according to the manufacturer’s instructions (bioMérieux).

### Antibiotic susceptibility testing.

The minimum inhibition concentration (MIC) was determined by using Etests and the standard agar dilution technique recommended by the CLSI using the supplemented Columbia agar (5% sheep blood, 1.0 g/L cellobiose, 1.0 g/L soluble starch, 1.0 g/L maltose, and 0.5 g/L l-cysteine, and with 10 mL/L hemin and 0.2 mL/L vitamin K1 solution added after autoclaving) instead of *brucella* agar. Columbia agar, which is considered comparable to *brucella* agar, was chosen as a substitute due to insufficient growth of *P. abscessus* CCUG 55929^T^ and *C. zoogleoformans* CCUG 20495^T^ (81, 82). Plates were inoculated with ca. 10^5^ CFU of each strain as recommended and cultivated for 48 h at 37°C in an anaerobic chamber with an Etest strip (Liofilchem) (83). Etests used for MICs determination were cefuroxime, clindamycin, erythromycin, metronidazole, penicillin G, and tetracycline. MICs were determined as the lowest concentration of antimicrobial agent resulting in growth inhibition. B. fragilis CCM 4712^T^ was used as a control strain.

### Analysis of end products of fermentation.

Fermentation end products were determined in modified reinforced clostridial medium (RCM) (Oxoid) with 0.5% glucose, after cultivation at 37°C for 4 days in an anaerobic chamber. Analysis was performed using capillary isotachophoresis (ITP) and the capillary electrophoretic analyzer Villa-Labeco EA 101 (Villa-Labeco, Slovakia) in capillary isotachophoretic mode (ITP) with the capillary of length 90 mm and diameter 0.8 mm. The initial voltage of 300 μA (290 s) was followed by 100 μA for separation as recommended by the manufacturer. The leading electrolyte comprised 10 mmol/L HCl, ε-aminocaproic acid (EACA) of pH 4.7 and 0.02% hydroxypropyl cellulose (HPC). The terminating electrolyte consisted of 10 mmol/L solution of caproic acid with histidine. The calibration standards included lactate, succinate, butyrate, propionate (QQ Standard Solutions for Ion Chromatography from Fluka), and 98% formate p.a. (Fluka), acetate p.a., and malate p.a. (both from Penta) diluted in Milli-Q water (Millipore Q GARD, Academic). The ITPwin and ITPpro software (Villa-Labeco) was used for final identification of fermentation end products.

### Chemotaxonomic analyses.

AN20^T^, AN421^T^, and AN502 strains were analyzed under the same conditions. The cultures were grown on modified peptone yeast glucose (PYG) medium (DSMZ, medium n.104) for 48 h at 37°C in anaerobic atmosphere until colonies reached the late exponential stage of growth according to the four-quadrant streak method (84). Extraction and subsequent analysis of fatty acid methyl esters were performed by Agilent 7890B gas chromatography according to the standard protocol of the Sherlock MIDI Identification System (MIDI Sherlock version 6.2, MIDI Database RTSBA 6.21). Analysis of respiratory quinones was carried out by the Identification Service of DSMZ (Braunschweig, Germany) using a freeze-dried biomass prepared from cells grown in modified PYG medium (DSMZ, medium n.104) cultivated for 1 to 3 weeks at 37°C.

### Data availability.

The GenBank accession numbers for whole-genome sequences of strains characterized in this study are as follows: BioSample SAMN06473662 (assembly accession number GCA_002160055.1) for AN20^T^, BioSample SAMN14565253 (assembly accession number GCA_019239235.1) for AN421^T^, and BioSample SAMN14565264 (assembly accession number GCA_019239315.1) for AN502. The GenBank accession numbers for the 16S rRNA gene sequences are MT894137 (AN20^T^), MT894142 (AN421^T^), and MT894135 (AN502).
