# Supplementary material for: Paraphocaeicola brunensis gen. nov., sp. nov., Carrying Two Variants of nimB Resistance Gene from Bacteroides fragilis, and Caecibacteroides pullorum gen. nov., sp. nov., Two Novel Genera Isolated from Chicken Caeca
Source: Microbiol Spectr. 2022 Feb 16;10(1):e01954-21. doi: 10.1128/spectrum.01954-21 (PMC8849064; doi:10.1128/spectrum.01954-21)
Supplement: SUPPLEMENTAL FILE 1 — Supplemental material. Download SPECTRUM01954-21_Supp_1_seq1.pdf, PDF file, 1.4 MB [file spectrum01954-21_supp_1_seq1.pdf]

Supplementary Material

Paraphocaeicola brunensis gen. nov., sp. nov., carrying two variants of *nimB* resistance gene from *Bacteroides fragilis*, and *Caecibacteroides pullorum* gen. nov., sp. nov., two novel genera isolated from chicken caeca.

Stanislava Kralova [1,2*], Lenka Davidova-Gerzova[1], Adam Valcek [3,4], Matej Bezdicek[5,6], Ivan Rychlik [7], Veronika Rezacova[8] and Alois Cizek [1,9]

1 - CEITEC VFU, University of Veterinary Sciences Brno, Brno, Czech Republic

2 - Department of Experimental Biology, Czech Collection of Microorganisms, Faculty of Science, Masaryk University, Brno, Czech Republic

3 - Microbial Resistance and Drug Discovery, VIB-VUB Center for Structural Biology, VIB, Flanders Institute for Biotechnology, Brussels, Belgium

4 - Structural Biology Brussels, Vrije Universiteit Brussel (VUB), Brussels, Belgium

5 – Department of Internal Medicine – Hematology and Oncology, University Hospital, Brno, Czech Republic

6 - Department of Internal Medicine – Hematology and Oncology, Masaryk University, Brno, Czech Republic

7 – Department of Bacteriology, Veterinary Research Institute, v. V. I., Brno, Czech Republic

8 - The Institute of Chemistry and Technology of Environmental Protection, Faculty of Chemistry, Brno University of Technology, Brno, Czech Republic

9 - Institute of Infectious Diseases and Microbiology, Faculty of Veterinary Medicine Brno, University of Veterinary Sciences Brno, Czech Republic

*** Correspondence:**

Stanislava Králová

kralova.s@sci.muni.cz

Keywords: phylogenomics, polyphasic taxonomy, *Paraphocaeicola brunensis* gen. nov., sp. nov., *Caecibacteroides pullorum* gen. nov., sp. nov., *Bacteroidaceae,* metronidazole resistance, *nimB* gene

Table S1. List of genomes of the closest related species from the family *Bacteroidaceae* retrieved from the NCBI database (https://www.ncbi.nlm.nih.gov/) used in the phylogenomic the analysis.

Species	Type strain number	Accession number
Bacteroides acidifaciens	JCM 10556^T	GCA_000613385.1
Bacteroides caccae	ATCC 43185^T	GCA_002222615.2
Bacteroides caecimuris	I48^T	GCA_001688725.1
Bacteroides cellulosilyticus	DSM 14838^T	GCA_000158035.1
Bacteroides clarus	YIT 12056^T	GCA_000195615.1
Bacteroides coprosuis	DSM 18011^T	GCA_000212915.1
Bacteroides egghertii	DSM 20669^T	GCA_000155815.1
Bacteroides faecalis	KCTC 15687^T	GCA_003865075.1
Bacteroides faecichinchillae	DSM 26883^T	GCA_900129065.1
Bacteroides faecis	MAJ27^T	GCA_000226135.2
Bacteroides finegoldii	DSM 17565^T	GCA_000156195.1
Bacteroides fluxus	YIT 12057^T	GCA_000195635.1
Bacteroides fragilis	NCTC 9343^T	GCA_000025985.1
Bacteroides gallinarum	DSM 18171^T	GCA_000374365.1
Bacteroides graminisolvens	DSM 19988^T	GCA_000428125.1
Bacteroides helcogenes	P 36-108^T	GCA_000186255.1
Bacteroides intestinalis	DSM 17393^T	GCA_000172175.1
Bacteroides koreensis	JCM 31393^T	GCA_007341375.1
Bacteroides kribbi	JCM 31391^T	GCA_007341395.1
Bacteroides luti	DSM 26991^T	GCA_900128905.1
Bacteroides nordii	WAL 11050^T	GCA_000613465.1
Bacteroides oleiciplenus	DSM 26884^T	GCA_900142015.1
Bacteroides ovatus	ATCC 8483^T	GCA_001314995.1
Bacteroides propionicifaciens	DSM 19291^T	GCA_000375405.1
Bacteroides pyogenes	DSM 20611^T	GCA_000428105.1
Bacteroides reticulotermitis	DSM 105720^T	GCA_014196955.1
Bacteroides rodentium	JCM 16496^T	GCA_000614125.1
Bacteroides salyersiae	WAL 10018^T	GCA_000381365.1
Bacteroides stercoris	DSM 19555^T	GCA_900106605.1
Bacteroides stercorisoris	DSM 26884^T	GCA_900142015.1
Bacteroides thetaiotaomicron	NCTC 10582^T	GCA_900445595.1
Bacteroides uniformis	ATCC 8492^T	GCA_000154205.1
Bacteroides xylanisolvens	XB1A^T	GCA_000210075.1
Capsularis zoogleoformans	ATCC 33285^T	GCA_002998435.1
Parabacteroides johnsonii	DSM 18315^T	GCA_000156495.1
Phocaeicola abscessus	CCUG 55292^T	GCA_000312445.1
Phocaeicola barnesiae	DSM 18169^T	GCA_000374585.1
Phocaeicola coprocola	DSM 17136^T	GCA_000154845.1
Phocaeicola coprophilus	DSM 18228^T	GCA_001315785.1
Phocaeicola dorei	DSM 17855^T	GCA_013009555.1
Phocaeicola massiliensis	DSM 17679^T	GCA_000382445.1
Phocaeicola paurosaccharolyticus	JCM 15092^T	GCA_000613805.1
Phocaeicola plebeius	DSM 17135^T	GCA_000187895.1
Phocaeicola salanitronis	DSM 18170^T	GCA_000190575.1
Phocaeicola sartorii	JCM 17136^T	GCA_000614185.1
Phocaeicola vulgatus	ATCC 8482^T	GCA_000012825.1

Table S2. 16S rRNA gene similaries and orthoANI values calculated between AN20^T, AN421^T, AN502 and the closest related species of the family *Bacteroidaceae*.

	16S rRNA gene similarities (%)			orthoANI values (%)		
	**AN20**	**AN421**	**AN502**	**AN20**	**AN421**	**AN502**
Bacteroides acidifaciens	89.03	91.29	91.15	70.78	71.33	71.15
Bacteroides caccae	88.61	90.95	90.88	70.22	70.90	70.81
Bacteroides caecimuris	88.61	91.23	90.67	70.84	71.36	71.18
Bacteroides cellulosilyticus	88.17	91.76	91.90	70.99	71.68	71.66
Bacteroides clarus	89.66	92.81	92.74	72.62	73.54	74.0
Bacteroides coprosuis	86.43	87.28	87.43	66.22	66.59	66.58
Bacteroides egghertii	89.78	93.43	93.50	72.43	73.63	73.3
Bacteroides faecalis	87.33	91.43	90.60	69.79	70.64	70.31
Bacteroides faecichinchillae	88.29	91.77	91.63	69.75	70.54	70.36
Bacteroides faecis	87.95	90.60	90.74	70.25	71.02	70.84
Bacteroides finegoldii	87.48	90.22	90.41	70.58	71.32	71.09
Bacteroides fluxus	89.15	91.43	91.29	72.13	73.29	73.04
Bacteroides fragilis	86.57	89.59	89.74	70.63	71.74	71.74
Bacteroides gallinarum	89.44	93.23	93.16	72.72	74.1	74.42
Bacteroides graminisolvens	87.65	90.67	90.59	68.64	69.42	69.23
Bacteroides helcogenes	89.70	91.70	91.63	72.0	73.13	73.15
Bacteroides intestinalis	88.90	91.42	91.56	71.0	71.83	71.61
Bacteroides koreensis	87.71	91.12	91.26	69.84	70.86	70.46
Bacteroides kribbi	86.97	90.42	90.49	69.86	70.81	70.46
Bacteroides luti	86.92	89.84	89.77	67.8	68.08	68.15
Bacteroides nordii	87.90	91.62	91.76	69.92	70.69	70.47
Bacteroides oleiciplenus	88.51	91.70	91.83	71.04	72.16	72.2
Bacteroides ovatus	87.68	90.00	90.21	70.13	70.73	70.51
Bacteroides propionicifaciens	85.27	86.91	86.92	66.02	66.49	66.42
Bacteroides pyogenes	88.05	89.67	89.61	70.6	71.6	71.53
Bacteroides reticulotermitis	88.18	90.54	90.61	69.4	70.12	70.04
Bacteroides rodentium	90.12	92.95	92.81	72.7	73.98	74.03
Bacteroides salyersiae	88.34	90.87	91.00	70.37	71.64	71.27
Bacteroides stercoris	89.26	91.91	91.85	71.31	72.46	72.35
Bacteroides stercorisoris	88.57	91.20	91.34	72.52	74.22	73.78
Bacteroides thetaiotaomicron	86.34	90.53	89.15	70.17	71.17	71.03
Bacteroides uniformis	90.34	93.23	93.09	72.38	73.97	73.54
Bacteroides xylanisolvens	88.08	90.69	90.91	70.37	70.93	70.96
Capsularis zoogleoformans	90.13	91.57	91.46	72.99	74.15	74.00
Phocaeicola abscessus	84.94	85.42	85.40	68.12	68.01	68.02
Phocaeicola barnesiae	88.76	90.95	90.81	70.85	71.64	70.85
Phocaeicola coprocola	89.89	91.57	91.71	70.04	70.41	70.04
Phocaeicola coprophilus	88.64	92.55	92.69	69.87	70.71	70.39
Phocaeicola dorei	89.58	90.36	90.50	69.61	69.73	69.9
Phocaeicola massiliensis	88.24	90.68	90.76	69.61	70.01	70.39
Phocaeicola paurosaccharolyticus	87.06	89.91	89.91	67.1	67.67	67.6
Phocaeicola plebeius	89.10	91.92	91.92	69.79	70.53	70.38
Phocaeicola salanitronis	89.72	90.23	90.16	73.28	71.01	70.63
Phocaeicola sartorii	88.57	90.95	90.95	69.5	70.08	70.08
Phocaeicola vulgatus	88.97	90.26	90.42	69.09	69.94	69.8

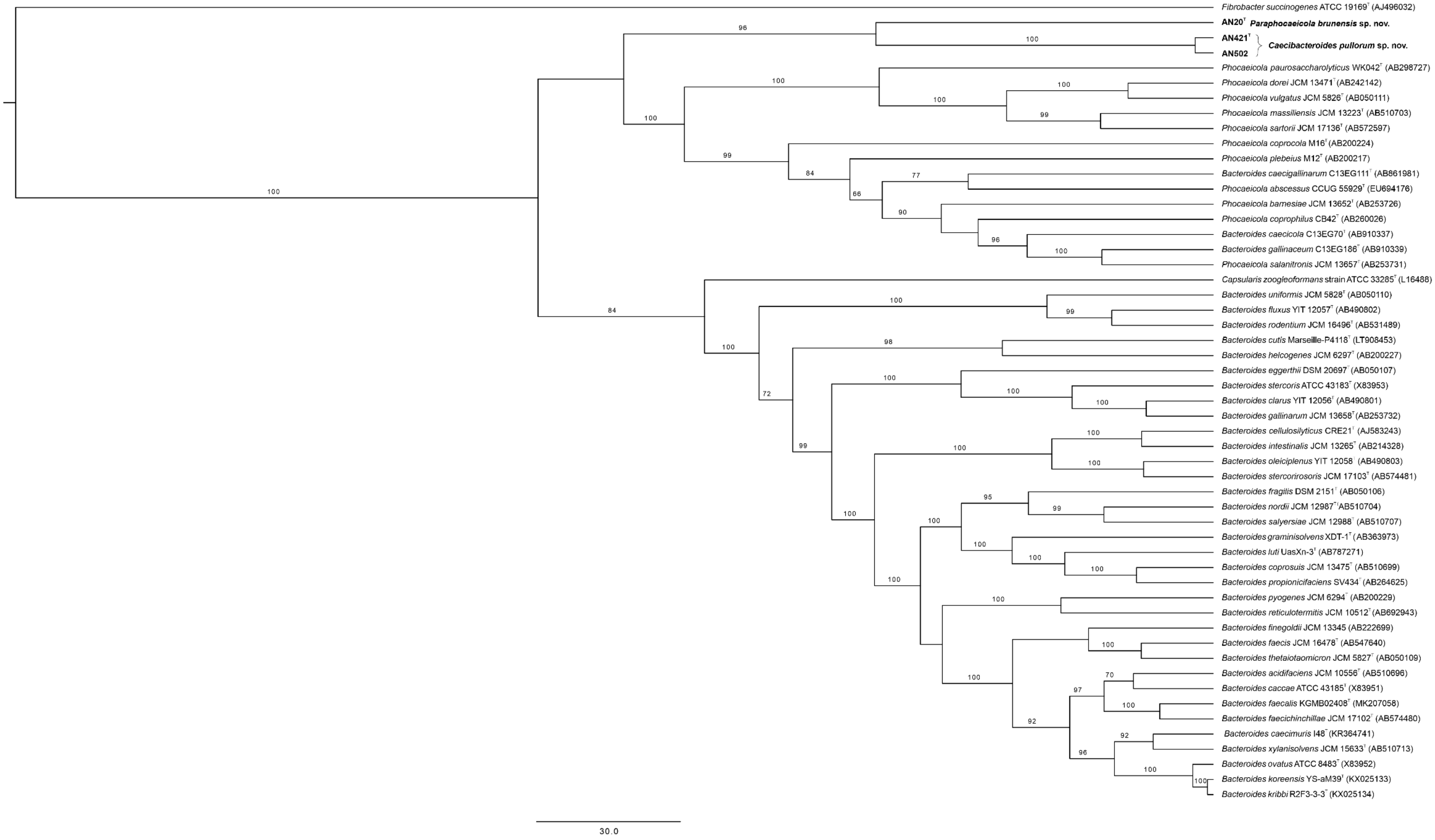

Figure S1. Bayesian phylogenetic tree obtained from analysis of the 16S rRNA gene sequence data. Statistical support for clades indicated by Bayesian posterior probabilities (multiplied by 100) are shown above the branches. The scale bar represents the number of nucleotide substitutions per site.

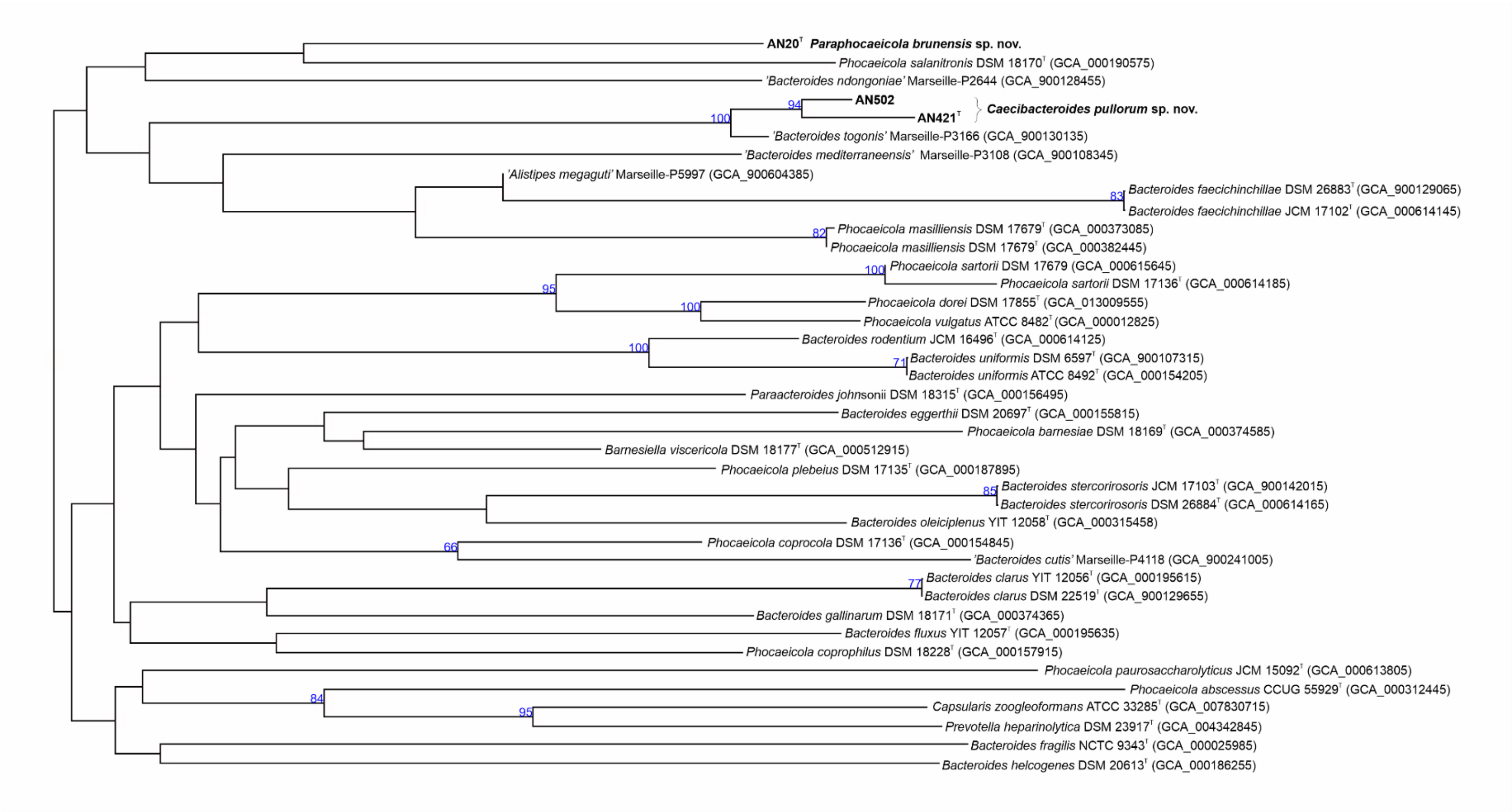

Figure S2. Phylogenomic trees obtained from the TYGS pair-wise comparison of AN20^T, AN421^T, AN502 and the closest related species determined by MASH algorithm. Phylogenomic tee was inferred with FastME v2.1.6.1 (Lefort et al., 2015). The numbers above the branches are GBDP (Genome-BLAST Distance Phylogeny) pseudo-bootstrap support values >60% from 100 replications, with an average branch support of 88.0 %.

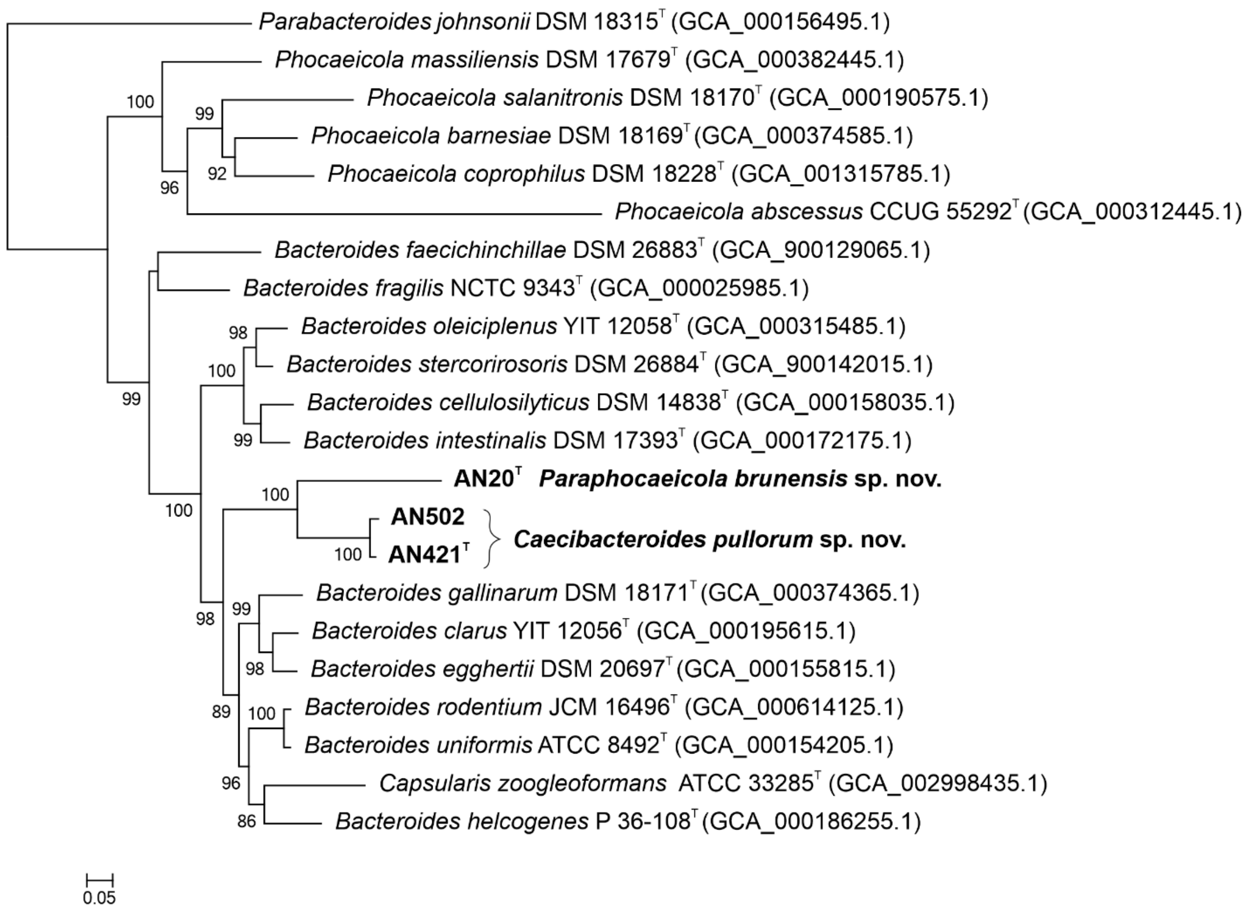

Figure S3. Phylogenetic tree highlighting the position of AN20^T, AN421^T and AN502 relative to the closest related species as determined by TYGS analysis. The whole genome SNP based phylogeny was established with CSI phylogeny 1.4 (Kaas et al., 2014) using the genome of *B. fragilis* NTCS 91343^T (GCA_000025985.1) as a reference and standard input parameters. The tree was visualized using FastTree v2.1 (Price et al., 2010). Only values > 60% are shown. The scale bar indicates the evolutionary distance between the sequences determined by 0.05 substitutions per nucleotide at the variable positions.

Table S3. Genome characteristics of *Paraphocaeicola brunensis* sp. nov. AN20^T and *Caecibacteroides pullorum* sp. nov. AN421^T and AN502.

	Strains		
	Paraphocaeicola brunensis sp. nov.	*Caecibacteroides pullorum* sp. nov.	
	AN20^T	AN421^T	AN502
WGS Project no.	NFJV00000000	JAAZTS000000000	JAAZTT000000000
Assembly method	IDBA-UD + SSPACE	IDBA-UD + SSPACE	IDBA-UD + SSPACE
Genome size (Mb)	3.84	3.61	3.87
Mean Coverage (×)	136	50	35
N50	119645	140168	80723
N75	63806	64604	51242
L50	12	8	16
L75	23	18	31
Largest contig (bp)	339547	463201	219944
No. of contigs > 200 bp	88	60	121
No. of contigs > 1000 bp	77	56	113
GC content (%)	49.52	48.66	48.35
Coding sequences	3247	2883	3127
No. of subsystems	ND	ND	ND
No. of RNAs	57	64	65
No. of tRNAs	55	59	62
No. of CRISPRs	1	1	4
No. of prophages	19	5	16
No. of plasmids	ND	ND	ND

Table S4. Number of genes associated with COGs functional categories.

COG class code	Description	AN20^T		AN421^T		AN502	
		Gene count	Percentage	Gene count	Percentage	Gene count	Percentage
	**Information storage and processing**						
A	RNA processing and modification	1	0,03%	1	0,03%	1	0,03%
J	Translation, ribosomal structure and biogenesis	159	4,83%	155	5,38%	154	4,92%
K	Transcription	166	5,04%	147	5,10%	163	5,21%
L	Replication, recombination and repair	235	7,13%	154	5,35%	187	5,98%
	**Metabolism**						
C	Energy production and conversion	134	4,07%	149	5,17%	145	4,63%
E	Amino acid transport and metabolism	180	5,46%	176	6,11%	175	5,59%
F	Nucleotide transport and metabolism	65	1,97%	76	2,64%	81	2,59%
G	Carbohydrate transport and metabolism	144	4,37%	216	7,50%	218	6,97%
H	Coenzyme transport and metabolism	111	3,37%	118	4,10%	120	3,84%
I	Lipid transport and metabolism	85	2,58%	76	2,64%	76	2,43%
P	Inorganic ion transport and metabolism	125	3,79%	146	5,07%	152	4,86%
Q	Secondary metabolites biosynthesis, transport, and catabolism	10	0,30%	17	0,59%	16	0,51%
	**Cellular processes and signaling**						
D	Cell cycle control, cell division, chromosome partitioning	36	1,09%	26	0,90%	35	1,12%
M	Cell wall/membrane/envelope biogenesis	247	7,50%	216	7,50%	227	7,25%
N	Cell motility	14	0,43%	30	1,04%	26	0,83%
O	Post-translational modification, protein turnover, and chaperones	64	1,94%	66	2,29%	66	2,11%
S	Function unknown	847	25,71%	561	19,47%	664	21,22%
T	Signal transduction mechanisms	57	1,73%	73	2,53%	78	2,49%
U	Intracellular trafficking, secretion, and vesicular transport	56	1,70%	25	0,87%	37	1,18%
V	Defense mechanisms	78	2,37%	82	2,85%	85	2,72%
	**Unknown category**						
	COG unknown	267	8,11%	191	6,63%	239	7,64%

Table S5. Genome comparison of AN20^T, AN421^T, AN502 and type strains of closely related genera.

Strain	Genome size (Mb)	G+C content (%mol)	Total gene count
AN20^T	3.84	49.5	3,386
AN421^T	3.61	48.7	3,008
AN502	3.87	48.4	3,246
B. fragilis CCM 4712^T	5.24	43.1	4,290
P. abscessus DSM 21584^T	2.54	47.2	2,766
C. zoogleoformans CCUG 20495^T	3.36	47.5	2,067

Table S6. CRISPR-Cas systems detected within genomes of AN20^{T}, AN421^{T} and AN502.

	CRISPR array N.	Scaffold	Position	Orientation	Spacer length	Repeat Length	No. of repeats	Repeat sequence (5´-3´)	adjacent *cas*-genes	Array family	Prediction confidence
AN20^{T}	CRISPR 1	6	84396-84551	forward	38	28	3	CTGTAAGGTCATTGTAAGATGTTGTAAG	no	not assigned	high

	CRISPR array N.	Scaffold	Position	Orientation	Spacer length	Repeat Length	No. of repeats	Repeat sequence (5´-3´)	adjacent cas-genes	Array family	Prediction confidence
AN421^{T}	CRISPR 1	1	40408-42529	reverse	30	47	28	GTTGTGATTTGCTTTCAAATTAGTATCTTTGAACCATTGGAGACAAC	yes	II-C	medium

	cas-genes	Scaffold	Position	COG number	Protein length (aa)	Accession N.	Description
AN421^{T}	*cas9*	1	34921-39033	COG3513	1370	MBV8057033.1	CRISPR/Cas system Type II associated protein, contains McrA/HNH and RuvC-like nuclease domains, Cas9
	cas1	1	39033-39968	COG1518	310	MBV8057034.1	CRISPR/Cas system-associated protein Cas1, endonuclease
	cas2	1	39995-40300	COG3512	111	MBV8057035.1	CRISPR/Cas system-associated protein Cas2, endoribonuclease

	CRISPR array N.	Scaffold	Position	Orientation	Spacer length	Repeat Length	No. of repeats	Repeat sequence (5´-3´)		Array family	Prediction confidence
AN502	CRISPR 1	13	1-661	forward	30	47	9	GTTGTGATTTGCTTTCAAATTAGTATCTTTGAACCATTGGAGACAAC	yes	II-C	high
	CRISPR 2	19	512-6	reverse	30	47	7	GTTGTGATTTGCTTTCAAATTAGTATCTTTGAACCATTGGAGACAAC	no	II-C	high
	CRISPR 3	106	360-5	reverse	30	47	5	GTTGTGATTTGCTTTCAAATTAGTATCTTTGAACCATTGGAGACAAC	no	II-C	high
	CRISPR 4	111	1049-6	reverse	30	47	14	GTTGTGATTTGCTTTCAAATTAGTATCTTTGAACCATTGGAGACAAC	no	II-C	high

	cas-genes	Scaffold	Position	COG number	Protein length (aa)	Accession N.	Description
AN502	*cas9*	13	2035-6144	COG3513	1396	MBV8039211.1	CRISPR/Cas system Type II associated protein, contains McrA/HNH and RuvC-like nuclease domains, Cas9
	cas1	13	768-1073	COG1518	310	MBV8039210.1	CRISPR/Cas system-associated protein Cas1, endonuclease
	cas2	13	1100-2032	COG3512	111	MBV8039209.1	CRISPR/Cas system-associated protein Cas2, endoribonuclease

Table S7. Putative resistance genes predicted in genomes of AN20^T, AN421^T and AN502.

	Gene	Scaffold	Position	Length (aa)	Coverage (%)	Identity (%)	Resistance Product	Protein Accession Number
AN20^T	*nimB*	scaffold 7	55323-55817	164	100.0	95.35	nitroimidazole resistance protein NimB	OUP09962.1
	nimB	scaffold 12	5611-6105	164	100.0	100.0	nitroimidazole resistance protein NimB	OUP08528.1
AN421^T	*lnuA$_{N2}$*	scaffold 23	16876-17388	170	100.0	99.81	lincosamide nucleotidyltransferase Lnu(AN2)	MBV8059400.1
	mefE$_{N2}$	scaffold 23	17413-18618	401	100.0	99.83	macrolide efflux MFS transporter Mef(En2)	MBV8059401.1
AN502	-	-	-	-	-	-	-	-

Table S8. Prevalence of highest 16S rRNA sequence matches to AN20^T, AN421^T and AN502 within metagenomes from the JGI IMG/M and GenBank databases.

Queried 16S rRNA sequence	Database		Length (bp)	16S rRNA gene similarity (%)	Isolation source	PUBMED	Where	Database ID/ Accession No.
AN20^T	JGI IMG/M	Metagenome analysis	731	99.0	Microbial communities from Fat line chicken, faeces	Unpublished	China	3300029905
	GenBank	Uncultured bacterium	1456	99.73	Intestinal microbiota of preadolescent turkeys, caecum	17284250	USA	DQ456092.1
			1455	99.45				DQ456050.1
			1453	99.11				DQ456084.1
			1434	99.72				DQ456033.1
AN421^{T}/AN502	JGI IMG/M	Metagenome analysis	1530	100.0/100.0	Human faecal microbial communities from infant at 12 months, faeces	34083435	Denmark	3300029180
	GenBank	Uncultured bacterium	1382	99.64/99.49	Mucosa-associated microbiota of inflamed and non-inflamed regions of the intestine in inflammatory bowel disease, colon	21219646	UK	FJ503680.1
			1442	99.51/99.93	Human ileum associated microbial composition in inflammatory bowel disease patients, ileum	22719818	USA	HQ792112.1
			1439	99.51/99.93				HQ792106.1
			1444	99.45/99.86				HQ792136.1
			1434	99.44/99.86				HQ792302.1
			1434	99.44/99.86				HQ792216.1

1487	99.39/99.80	Faecal community in humans, faeces	Unpublished	Canada	GQ897811.1
1447	99.38/99.79	Human ileum associated microbial composition in inflammatory bowel disease patients, ileum	22719818	USA	HQ792094.1
1447	99.31/99.72				HQ792211.1
1447	99.10/99.52				HQ792200.1
1446	99.10/99.52				HQ792227.1
1445	99.10/99.51				HQ792179.1
1442	99.10/99.51				HQ792115.1
1441	99.10/99.51				HQ792205.1
1437	99.10/99.51				HQ792243.1
1445	99.03/99.45				HQ792270.1
1441	99.03/99.44				HQ792162.1
1382	98.99/99.13	Mucosa-associated microbiota of inflamed and non-inflamed regions of the intestine in inflammatory bowel disease, colon	21219646	UK	FJ503772.1
1451	98.97/98.97	Intestinal microbiota of preadolescent turkeys, caecum	17284250	USA	DQ456088.1
1447	98.96/99.38	Human ileum associated microbial composition in inflammatory bowel disease patients, ileum	22719818	USA	HQ791921.1
1382	98.92/99.06	Mucosa-associated microbiota of inflamed and non-inflamed regions of the intestine in inflammatory bowel disease, colon	21219646	UK	FJ503894.1
1452	98.83/99.24	Intestinal microbiota of preadolescent turkeys, caecum	17284250	USA	DQ456007.1
1443	98.82/99.24	Human ileum associated microbial composition in inflammatory bowel disease patients, ileum	22719818	USA	HQ792228.1
1440	98.82/99.24				HQ792257.1
1442	98.82/99.24				HQ792309.1
1438	98.82/99.24				HQ792149.1
1443	98.75/99.17				HQ791844.1
1440	98.75/99.17				HQ792254.1
1439	98.75/99.17				HQ792313.1
1450	98.55/98.97	Caecal microbiota of domestic and wild turkeys	18183454	USA	EU009790.1
1444	98.27/98.69	Human ileum associated microbial composition in inflammatory bowel disease patients, ileum	22719818	USA	HQ792154.1

Table S9: Formal descriptions of *Paraphocaeicola brunensis* sp. nov. and *Caecibacteroides pullorum* sp. nov.

**Genus name**	*Paraphocaeicola* gen. nov.	*Caecibacteroides* gen. nov.
**Species name**	*Paraphocaeicola brunensis*	*Caecibacteroides pullorum*
**Specific epithet**	*brunensis*	*pullorum*
**Species status**	sp. nov.	sp. nov
**Species etymology**	bru.nen'sis. L. adj. *brunensis* from *Bruna*, the Roman name of the city of Brno, Czech Republic, where the type strain was isolated	pul.lo'rum. L. gen. pl. n. *pullorum*, chicken; referring to the source of isolation
**Description of the new taxon and diagnostic traits**	**Phenotypic characteristics:**<br>• Gram-negative rods with rounded ends, size 0.8-0.9 x 1.8-20.0 µm<br>• greyish white, circular, entire, low convex colonies on WCHA agar plates, diameter 1.5-1.7 mm<br>• haemolysis on Columbia agar after 48 hrs<br>• does not adhere to agar<br>• temperature range 28-47 °C, optimum 37°C<br>• pH range 6-12.0, optimum 7.0<br>• grows at 0.5-1.5 % NaCl, 2.0 % NaCl inhibits growth<br>• resistant to bile acids<br>• no growth in microaerophilic conditions<br>• catalase and oxidase negative<br>• growth observed on WCHA, WCHA supplemented with rumen fluid, Columbia agar<br>• negative for production of urease and indole<br>• negative for reduction of nitrates and nitrites<br>• hydrolyses gelatine and cellulose (weakly)<br>• no hydrolysis of aesculine and carboxymethylcellulose<br>• RAPID 20A negative: indole, urease, mannitol, D-saccharose, salicin, D-xylose, L-arabinose, esculin, glycerol, D-cellobiose, D-melezitose, D-raffinose, D-sorbitol, L-rhamnose, D-trehalose<br>• RAPID 20A positive: D-glucose (weakly), D-lactose, D-maltose, gelatine, D-mannose<br>• API RAPID 32A negative: urease, arginine dihydrolase, β-galactosidase-6-phosphate, α-glucosidase, β-glucosidase, α-arabinosidase, β-glucuronidase, D-mannose and D-raffinose	**Phenotypic characteristics:**<br>• Gram-negative rods with rounded ends, size 0.4-1.2 x 0.6-24.0 µm<br>• greyish white, circular, entire, low convex colonies on WCHA agar plates, diameter 1.0 mm<br>• haemolysis on Columbia agar after 96 hrs<br>• does not adhere to agar<br>• temperature range 30-40 °C, optimum 37°C<br>• pH range 6-11.0, optimum 7.0<br>• grows at 0.0-0.5 % NaCl, 1.0 % NaCl inhibits growth<br>• sensitive to bile acids<br>• no growth in microaerophilic conditions<br>• catalase and oxidase negative<br>• growth observed on WCHA, WCHA supplemented with rumen fluid, Columbia agar<br>• negative for production of urease and indole<br>• hydrolyses gelatine<br>• no hydrolysis of aesculine, cellulose and carboxymethylcellulose<br>• RAPID 20A negative: indole, urease, mannitol, D-saccharose, salicin, glycerol, D-cellobiose, D-melezitose, D-raffinose, D-sorbitol, D-trehalose<br>• RAPID 20A positive: D-glucose, D-lactose, D-saccharose, D-maltose, gelatine, D-mannose, D-raffinose<br>• API RAPID 32A negative: urease, arginine dihydrolase, β-galactosidase-6-phosphate, β-glucosidase, α-arabinosidase, β-glucuronidase, mannose fermentation, glutamic acid decarboxylase, reduction of nitrates, production of indole, proline

	fermentation, glutamic acid decarboxylase, reduction of nitrates, production of indole, arginine arylamidase, proline arylamidase, phenylalanine arylamidase, leucine arylamidase, pyroglutamic acid arylamidase, tyrosine arylamidase, glycine arylamidase, histidine arylamidase, glutamyl glutamic acid arylamidase, serine arylamidase<br>• API RAPID 32A positive: α-galactosidase, β-galactosidase, N-acetyl-β-glucosaminidase, α-fucosidase, leucyl glycine arylamidase, alanine arylamidase	arylamidase, pyroglutamic acid arylamidase, tyrosine arylamidase, glycine arylamidase, histidine arylamidase, glutamyl glutamic acid arylamidase, serine arylamidase<br>• API RAPID 32A positive: α-galactosidase, β-galactosidase, α-glucosidase, N-acetyl-β-glucosaminidase, D-raffinose fermentation, α-fucosidase, alkaline phosphatase, arginine arylamidase, leucyl glycine arylamidase, phenylalanine arylamidase, leucine arylamidase, alanine arylamidase
	**Chemotaxonomic characteristics:**<br>• major fatty acids: $C_{18:1}$ *ω9c*, iso-$C_{17:0}$ 3OH, iso- $C_{15:0}$, anteiso-$C_{15:0}$, $C_{16:0}$<br>• major respiratory quinone are MK-5 and MK-11	**Chemotaxonomic characteristics:**<br>• major fatty acids: $C_{18:1}$ *ω9c*, iso-$C_{17:0}$ 3OH, $C_{16:0}$ 3OH, iso- $C_{15:0}$, anteiso-$C_{15:0}$ and $C_{16:0}$<br>• major respiratory quinone are MK-5 and MK-10
**Type Strain Information**		
**Country of origin**	Czech Republic	Czech Republic
**Region of origin**	Brno	Brno
**Date of isolation**	15.10.2015	01.03.2017
**Source of isolation**	chicken (Lohman Brown Light variety), caecum	chicken (Lohman Brown Light variety), caecum
**Sampling date**	08.10.2015	23.02.2017
**Latitude**	49° 11' 42.79" N	49° 11' 42.79" N
**Longitude**	16° 36' 28.66" E	16° 36' 28.66" E
**Altitude**	237m	237m
**16S rRNA gene accession nr.**	MT894137	MT894142
**Genome accession number**	GCA_002160055.1	GCA_019239235.1
**Genome status**	incomplete	incomplete
**Genome size**	3,84 Mbp	3,74 Mbp
**GC mol%**	49.52	48.66
**Number of strains in study**	1	2
**Source of isolation of non-type strains**	chicken caecum	chicken caecum
**Designation of the Type Strain**	AN20^T = CCM 9041^T = DSM 111154^T	AN421^T = CCM 9040^T = DSM 111155^T

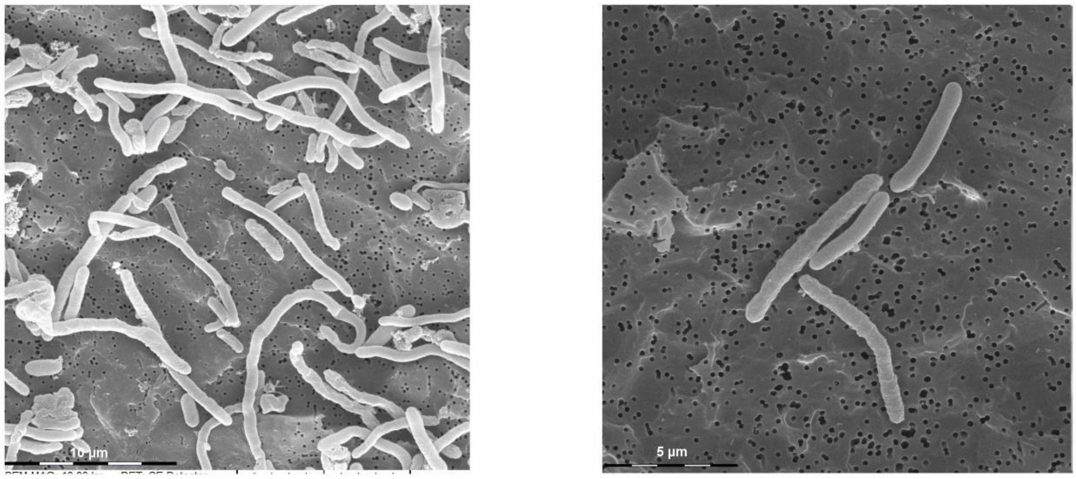

Figure S4. Cellular morphology of strain *Paraphocaeicola brunensis* sp. nov. AN20^{T}. Images were obtained using by scanning electron microscopy (SEM) performed with Tescan Vega scanning electron microscope (Czech Republic) after coating with 10 nm gold/palladium.

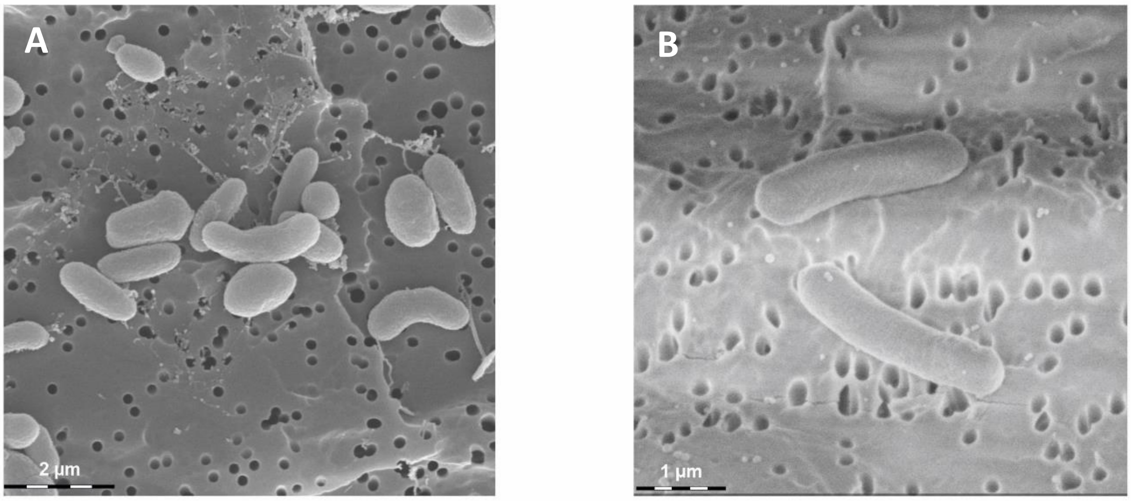

Figure S5. Cellular morphology of strain *Caecibacteroides pullorum* sp. nov. (A) AN421^{T} and (B) AN502. Images were obtained using by scanning electron microscopy (SEM) performed with Tescan Vega scanning electron microscope (Czech Republic) after coating with 10 nm gold/palladium.

References:

Kaas, R. S., Leekitcharoenphon, P., Aarestrup, F. M., and Lund, O. (2014). Solving the Problem of Comparing Whole Bacterial Genomes across Different Sequencing Platforms. *PLOS ONE* 9, e104984. doi:10.1371/journal.pone.0104984.

Lefort, V., Desper, R., and Gascuel, O. (2015). FastME 2.0: A Comprehensive, Accurate, and Fast Distance-Based Phylogeny Inference Program. *Mol Biol Evol* 32, 2798–2800. doi:10.1093/molbev/msv150.

Price, M. N., Dehal, P. S., and Arkin, A. P. (2010). FastTree 2 – Approximately Maximum-Likelihood Trees for Large Alignments. *PLOS ONE* 5, e9490. doi:10.1371/journal.pone.0009490.
